# HiBiT-qIP, HiBiT-based quantitative immunoprecipitation, facilitates the determination of antibody affinity under immunoprecipitation conditions

**DOI:** 10.1038/s41598-019-43319-y

**Published:** 2019-05-03

**Authors:** Deshani C. Ranawakage, Takuya Takada, Yusuke Kamachi

**Affiliations:** 1 School of Environmental Science and Engineering, Kochi University of Technology, 185 Miyanokuchi, Tosayamada-cho, Kami, Kochi 782-8502 Japan; 2 Graduate School of Frontier Biosciences, Osaka University 1–3 Yamadaoka, Suita, Osaka 565-0871 Japan

**Keywords:** Proteins, Biochemical assays, Immunoprecipitation

## Abstract

The affinity of an antibody for its antigen serves as a critical parameter for antibody evaluation. The evaluation of antibody-antigen affinity is essential for a successful antibody-based assay, particularly immunoprecipitation (IP), due to its strict dependency on antibody performance. However, the determination of antibody affinity or its quantitative determinant, the dissociation constant (K_d_), under IP conditions is difficult. In the current study, we used a NanoLuc-based HiBiT system to establish a HiBiT-based quantitative immunoprecipitation (HiBiT-qIP) assay for determining the K_d_ of antigen-antibody interactions in solution. The HiBiT-qIP method measures the amount of immunoprecipitated proteins tagged with HiBiT in a simple yet quantitative manner. We used this method to measure the K_d_ values of epitope tag-antibody interactions. To accomplish this, FLAG, HA, V5, PA and Ty1 epitope tags in their monomeric, dimeric or trimeric form were fused with glutathione S-transferase (GST) and the HiBiT peptide, and these tagged GST proteins were mixed with cognate monoclonal antibodies in IP buffer for the assessment of the apparent K_d_ values. This HiBiT-qIP assay showed a considerable variation in the K_d_ values among the examined antibody clones. Additionally, the use of epitope tags in multimeric form revealed a copy number-dependent increase in the apparent affinity.

## Introduction

A broad range of research, diagnostic and therapeutic activities are inseparably linked to the use of antibodies for the enrichment, detection and quantitation of proteins and their modifications. The success of these procedures is highly dependent on the quality of the antibodies, which is critically determined by the affinity and specificity of the antibodies towards their cognate antigens. Although there are hundreds of thousands of commercially available antibodies, many of them have been poorly characterised and are thus inadequately reliable, which makes it difficult to find a suitable antibody for a specific application^1–6^. Immunoprecipitation (IP) is an immunological technique in which specific antibodies are used to enrich the target proteins or protein complexes from a protein mixture solution. IP has been extensively applied in many scientific fields to identify and study protein-protein and protein-DNA interactions^7^. Stringent assessment of the degree of sensitivity and specificity of an antibody in capturing its cognate antigen is required for a successful IP assay, particularly chromatin immunoprecipitation (ChIP)^2,8^.

The sensitivity of an antibody-based assay is essentially determined by the binding affinity of the antibody to its cognate antigen. Thus, the measurement of the affinity of an antibody can predict its suitability for future IP experiments in advance. The dissociation constant (K_d_) of an antigen-antibody interaction quantitatively defines its binding affinity. The most popular and widely used methods for determining K_d_ include enzyme-linked immunosorbent assay (ELISA)-based methods^9^, surface plasmon resonance (SPR) biosensors^10,11^ and the solution-based kinetic exclusion assay (KinExA)^12,13^. Each of these methods has its own inherent advantages and disadvantages^7,12,14^, and different K_d_ values can be obtained by the different types of immunological assays, as is described in the literature^12,15^. Because none of these methods reflects the IP protocol, it is difficult to predict the performance of an antibody during actual IP experiments, where antibody-antigen reactions are influenced by many factors. The critical parameters that affect the equilibrium constant are the ionic strength, pH, temperature, and the composition of ionic and nonionic detergents in the IP buffer^7,16,17^. Thus, determining apparent K_d_ values under particular IP conditions would be desirable. However, determining the K_d_ values of an antibody under IP conditions is challenging, mainly because the amount of precipitated protein is often near or below the lowest limit of quantitative detection by Western blotting, which is usually in the range of hundreds of picograms^18,19^.

In this manuscript, we report a simple and relatively inexpensive approach for determining the antibody K_d_ under IP conditions by employing a quantitative NanoLuc-based HiBiT detection system. We call this method HiBiT-qIP, which is short for “HiBiT-based quantitative immunoprecipitation”. The HiBiT system is based on the split luciferase complementation of two NanoLuc fragments. Specifically, a 1.3-kDa peptide (11 amino acids) is capable of producing bright luminescence through interaction with an 18-kDa polypeptide named Large BiT (LgBiT). During the development of the split luciferase complementation assay, a small peptide, which is called Small BiT (SmBiT) and has low affinity (K_d_ > 100 µM) to LgBiT, was initially adopted for the accurate measurement of protein interaction within cells^20^. In contrast, in the newly developed HiBiT system, the high-affinity (K_d_ = 0.7 nM) binding of a novel 11-amino acid High BiT (HiBiT) peptide to LgBiT efficiently forms a stable complex that acts as the active NanoLuc luciferase, which enables HiBiT to serve as a quantitative luminescent peptide tag^20,21^. Thus, tagging a protein of interest with the HiBiT peptide facilitates sensitive quantification of the amount of HiBiT-tagged protein, which makes it possible to measure protein amounts of less than 1 amol (e.g., 0.05 pg of a 50-kDa protein)^21–23^. Furthermore, a simple add-mix-read assay protocol of the HiBiT detection system enabled us to perform the IP-based equilibrium binding analysis more easily.

In the current study, we applied the HiBiT-qIP method to evaluate monoclonal antibodies against epitope tags that are widely utilised in immunoprecipitation. Epitope tagging of a target protein with a short peptide and subsequent use of epitope-specific antibodies to immunoprecipitate the tagged protein is a promising strategy for circumventing the lack of antibodies against target proteins^24–27^. This strategy is gaining popularity because an epitope tag can now be introduced into an endogenous target protein by adapting the clustered regularly interspaced short palindromic repeats (CRISPR)/Cas9 genome editing tool to express the tagged proteins at near-endogenous levels^27–30^. Here, we examined the affinities of monoclonal antibodies against the epitope tags of FLAG^31,32^, HA^33^ and V5^34^, PA^35^ and Ty1^36^ because little information on their K_d_ values is currently available in spite of their widespread usage^26,37^. The PA tag was examined because it was recently reported that the rat monoclonal antibody NZ-1 against human podoplanin can be used as a high-affinity tagging system^35^. The Ty1 tag reportedly exhibits high performance in ChIP and was thus also included in our analysis^38^.

The performance of an epitope tag in an IP experiment depends not only on the amino acid sequence of the epitope tag used but also substantially on the quality of the anti-epitope tag antibody. Furthermore, a number of monoclonal clones for some epitope tags, such as FLAG, have been developed and are commercially available. Therefore, the selection of the optimal epitope tag/antibody combination is a prerequisite for truly successful IP experiments, but such selection has rarely been performed. Moreover, epitope tags have often been used as multimerised forms to increase the efficiency of IP experiments, but their effects have not been quantitatively studied. Here, we aimed to evaluate the optimal epitope tag/antibody combinations suitable for IP and the effects of tag multimerisation by developing the HiBiT-based quantitative immunoprecipitation assay (HiBiT-qIP) and using this assay to determine the apparent K_d_ values of various combinations. As we compared a collection of epitope tags in combination with commercially available monoclonal antibodies, the findings of this study will constitute a valuable resource for future IP-related experiments.

## Results

### Design of an assay for the determination of antibody affinity using the HiBiT system

The NanoLuc-based HiBiT system is an accurate and sensitive protein quantification approach due to the linearity and stability of the luminescence signal generated by HiBiT/LgBiT, namely, the reconstituted NanoLuc luciferase^21^. By tagging a protein of interest with the HiBiT peptide, its amount can be easily and accurately quantified using the HiBiT detection reagent containing LgBiT and the luciferase substrate furimazine^21–23^. Employing this system, we designed an assay to evaluate the suitability of an antibody for IP by determining the antibody dissociation constant K_d_ under specific IP reaction conditions. In the current study, we applied the HiBiT-qIP assay to determine the K_d_ values of monoclonal antibodies against the epitope tags FLAG, HA, V5, PA and Ty1, which were selected based on their wide usage in research and the commercial availability of corresponding monoclonal antibodies (Tables 1 and 2).

**Table 1:** Evaluated epitope tag sequences.

Epitope tag	Sequence
FLAG	**DYKDDDDK**
FLAGx3	**DYKDDDDK**G**DYKDDDDK**I**DYKDDDDK**
HA	**YPYDVPDYA**
HAx3	**YPYDVPDYA**G**YPYDVPDYA**G**YPYDVPDYA**
V5	**GKPIPNPLLGLDST**
V5x2	**GKPIPNPLLGLDST**G**GKPIPNPLLGLDST**
V5x3	**GKPIPNPLLGLDST**G**GKPIPNPLLGLDST**G**GKPIPNPLLGLDST**
PA	**GVAMPGAEDDVV**
PAx2	**GVAMPGAEDDVV**G**GVAMPGAEDDVV**
PAx3	**GVAMPGAEDDVV**G**GVAMPGAEDDVV**TR**GVAMPGAEDDVV**
Ty1	**EVHTNQDPLD**
Ty1x2	**EVHTNQDPLD**A**EVHTNQDPLD**
Ty1x3	**EVHTNQDPLD**A**EVHTNQDPLD**TR**EVHTNQDPLD**

**Table 2:** Detailed overview of characterised antibodies.

Antibody type	Supplier	Clone	Species	IgG subclass	Bead type^a^	Tag	K_d_ (nM)	95% Confidence interval of K_d_ (nM)
Anti-FLAG	Sigma	M2	Mouse	IgG1	α-mouse IgG beads	FLAG	0.76^b^	0.44–1.3
						FLAGx3	0.21	0.12–0.37
	Wako	IE6	Mouse	IgG2b	α-mouse IgG beads	FLAG	1.8	1.3–2.6
						FLAGx3	0.50	0.40–0.62
	MBL	FLA-1	Mouse	IgG2a κ	α-mouse IgG beads	FLAG	1.3	0.82–2.0
						FLAGx3	0.33	0.22–0.50
	BioLegend	L5	Rat	IgG2a	α-rat IgG beads	FLAG	0.44	0.27–0.72
						FLAGx3	0.16	0.097–0.20
Anti-HA	Roche	3F10	Rat	IgG1	α-rat IgG beads	HA	0.38	0.22–0.70
						HAx3	0.067	0.035–0.12
	Wako	4B2	Mouse	IgG2b	α-mouse IgG beads	HA	6.6^b^	5.5–8.0
						HAx3	0.88	0.50–1.5
Anti-V5	Sigma	V5-10	Mouse	IgG1	α-mouse IgG beads	V5	0.59	0.37–0.95
						V5x2	0.36	0.22–0.58
						V5x3	0.23	0.15–0.35
	Wako	6F5	Mouse	IgG2b	α-mouse IgG beads	V5	0.42	0.22–0.77
						V5x2	0.28	0.16–0.47
						V5x3	0.28	0.18–0.44
Anti-PA	Wako	NZ-1	Rat	IgG2a	α-rat IgG beads	PA	0.65^b^	0.38–1.1
						PAx2	0.38	0.26–0.54
						PAx3	0.27	0.17–0.42
Anti-Ty1	Sigma	BB2	Mouse	IgG1	α-mouse IgG beads	Ty1	0.39^b^	0.22–0.65
						Ty1x2	0.29	0.17–0.48
						Ty1x3	0.20	0.12–0.34

^a^Anti-IgG magnetic beads used to capture the antibodies.

^b^K_d_ values obtained by combining data from two independent experiments.

**Figure 1:**
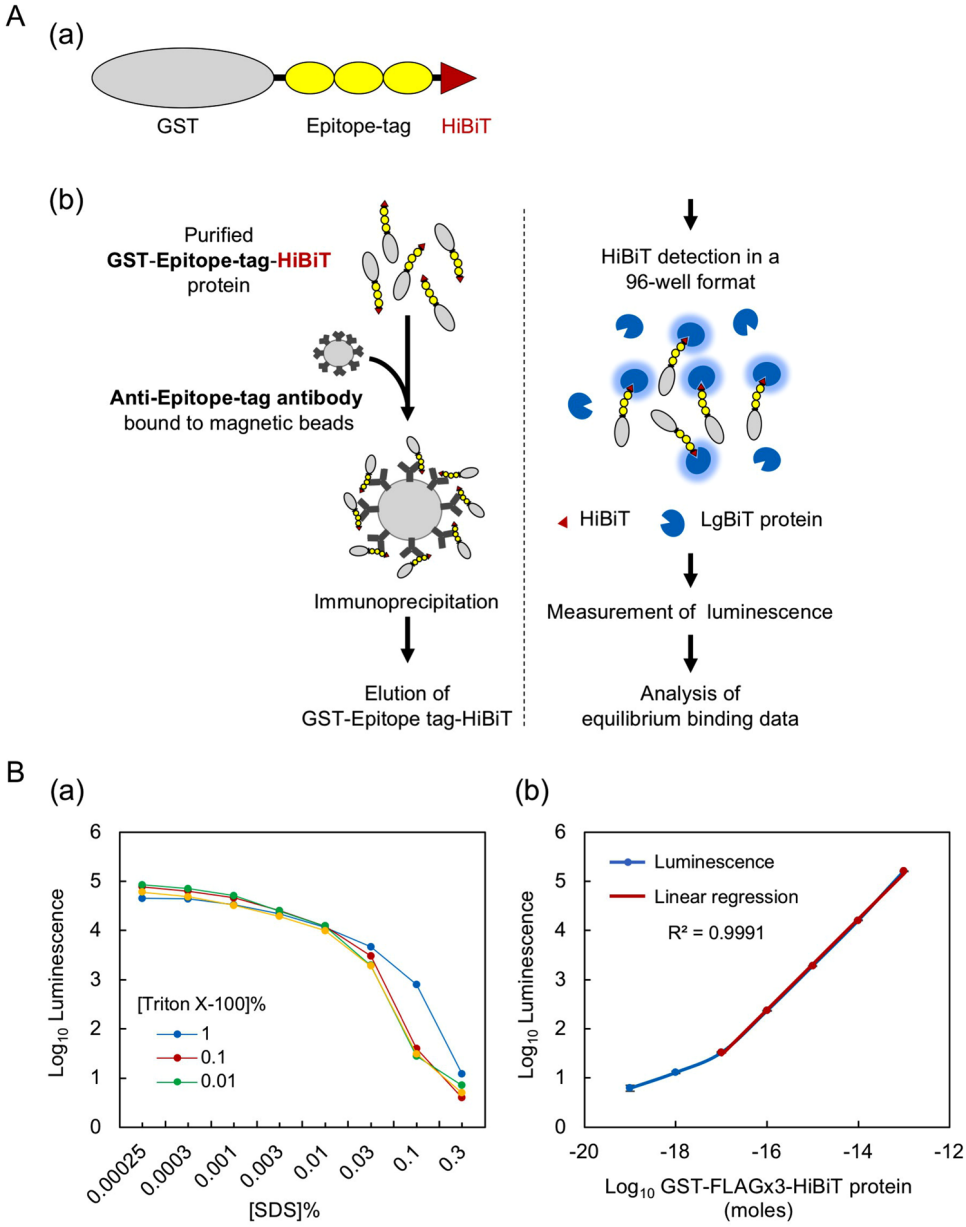
HiBiT-based quantitative immunoprecipitation. (**A**) Design of the assay. (a) Schematic representation of the GST-epitope tag-HiBiT fusion protein. The coding region of the GST gene is C-terminally fused to the FLAG, HA, V5, PA or Ty1 epitope tags in their monomeric, dimeric or trimeric form and the HiBiT peptide, which is placed in the most C-terminal position. In this panel, the trimeric form of the epitope tags is shown as an example; the tags are not drawn to scale. (b) Illustration showing the main steps of the HiBiT-qIP assay and the principle of HiBiT detection. The details are provided in the main text. (**B**) HiBiT protein quantitation in the presence of SDS. (a) Effect of SDS and Triton X-100 on the HiBiT solution assay. To examine the effects of SDS on the enzymatic activity of reconstituted NanoLuc, 0.2 ng of GST-FLAGx3-HiBiT protein was included in 20 µL of PBS containing one of a series of concentrations of SDS (0.00025 to 0.3%), and the luminescence was measured after the addition of HiBiT detection reagents. The optimal Triton X-100 concentration for quenching the SDS effect was determined by adding Triton X-100 at three different concentrations, as indicated. (b) Linearity of the luminescence generated by HiBiT-LgBiT under our assay conditions. A tenfold dilution series of GST-FLAGx3-HiBiT protein (3.3 fg [10^−19^ moles] to 3.3 ng [10^−13^ moles]) in 20 µL of PBS containing 0.001% SDS, 0.01% BSA and 0.1% Triton X-100 was used in the HiBiT solution assay.

These epitope tags, in their monomeric, dimeric (x2) or trimeric (x3) form, were first fused to the GST protein and the HiBiT peptide (Fig. 1Aa, Supplementary Fig. 1, Table 1). The epitope-tagged GST proteins were then expressed in and purified from *E. coli* to near homogeneity. The purified proteins were separated using SDS-polyacrylamide gels, stained with Coomassie Brilliant Blue G-250 and quantified based on the infrared fluorescence of Coomassie blue^39^ (Supplementary Fig. 2A,B). We confirmed the full-length protein bands using the Nano-Glo HiBiT Blotting System^22,23^ (Supplementary Fig. 2C) and quantified only the intact proteins.

Varying amounts of the purified epitope-tagged GST protein were then mixed with a fixed amount of cognate monoclonal antibody immobilised on anti-IgG magnetic beads in a stringent IP buffer, which has been extensively used as the buffer for radio-immunoprecipitation assays (RIPAs)^7,40,41^. Importantly, the amount of antibody used during IP was optimised to maintain the concentration close to, or lower than, the K_d_ of each antibody, as suggested for standard binding assays^42^. The IP mixtures were incubated overnight at 4 °C, during which time the binding reaction between the antigen and antibody was assumed to reach equilibrium because most IP reactions reportedly reach the plateau phase within a few hours^16,43,44^. After overnight incubation of the IP mixtures, the unbound proteins were washed away, and the amount of bound epitope-tagged GST protein was determined by measuring the luminescence signal derived by the HiBiT/LgBiT complex (Fig. 1Ab). A saturation curve of bound GST as a function of free GST was plotted by fitting the data to the binding model mentioned in the methodology section, and the K_d_ values were determined. For all K_d_ determinations, error graphs were plotted, and the 95% confidence intervals were calculated. We consider the obtained K_d_ values as “apparent” K_d_ values under our IP conditions. The “apparent” K_d_ values take into consideration factors such as antibody valency, steric hindrance and the mode of antibody immobilisation^45,46^. The apparent K_d_ values thus may not be identical to true K_d_ values that would be obtained through an ideal assay using a completely homogeneous solution.

### HiBiT protein quantitation can be performed in the presence of residual SDS

When using the HiBiT system for IP experiments, one should consider the effect of the residual SDS derived from the IP elution buffer on the interaction between HiBiT and LgBiT. Therefore, we first examined the effects of SDS on the HiBiT solution assay by measuring the luminescence values in the presence of varying concentrations of SDS in the sample solution. We also sought to determine the optimal concentration of Triton X-100 that could effectively quench the disruptive effect of SDS. When we used 0.2 ng of the purified GST-FLAGx3-HiBiT protein in the HiBiT solution assay, SDS clearly inhibited the interaction between HiBiT and LgBiT, even at low concentrations (Fig. 1Ba). The results also showed that 1% Triton X-100 exerted an SDS-quenching effect in the presence of >0.01% SDS, as expected, but slightly inhibited the HiBiT solution assay in the presence of <0.01% SDS. At these lower SDS concentrations, moderate concentrations of Triton X-100 exhibited the SDS-quenching effect. Based on these observations, we adjusted the final SDS concentration to 0.001% and added 0.1% Triton X-100 to the assay samples in the subsequent experiments. In addition, to minimise the SDS concentration in the assay samples, we used IP elution buffer containing 0.1% SDS and 25 mM DTT.

We then confirmed the linearity of the luminescence generated by HiBiT/LgBiT under the above conditions. Specifically, a tenfold dilution series was prepared starting from 3.3 ng of GST-FLAGx3-HiBiT with phosphate buffered saline (PBS) containing 0.01% bovine serum albumin (BSA) in addition to 0.1% TritonX-100 and 0.001% SDS. In the presence of saturating LgBiT in the HiBiT assay reagent solution, GST-FLAGx3-HiBiT produced luminescent signals that were linearly correlated to the protein amounts (shown in red line in Fig. 1Bb), with a lower limit of approximately 0.33 pg (0.01 fmol).

### The K_d_ values varied considerably among monoclonal antibody clones

**Figure 2:**
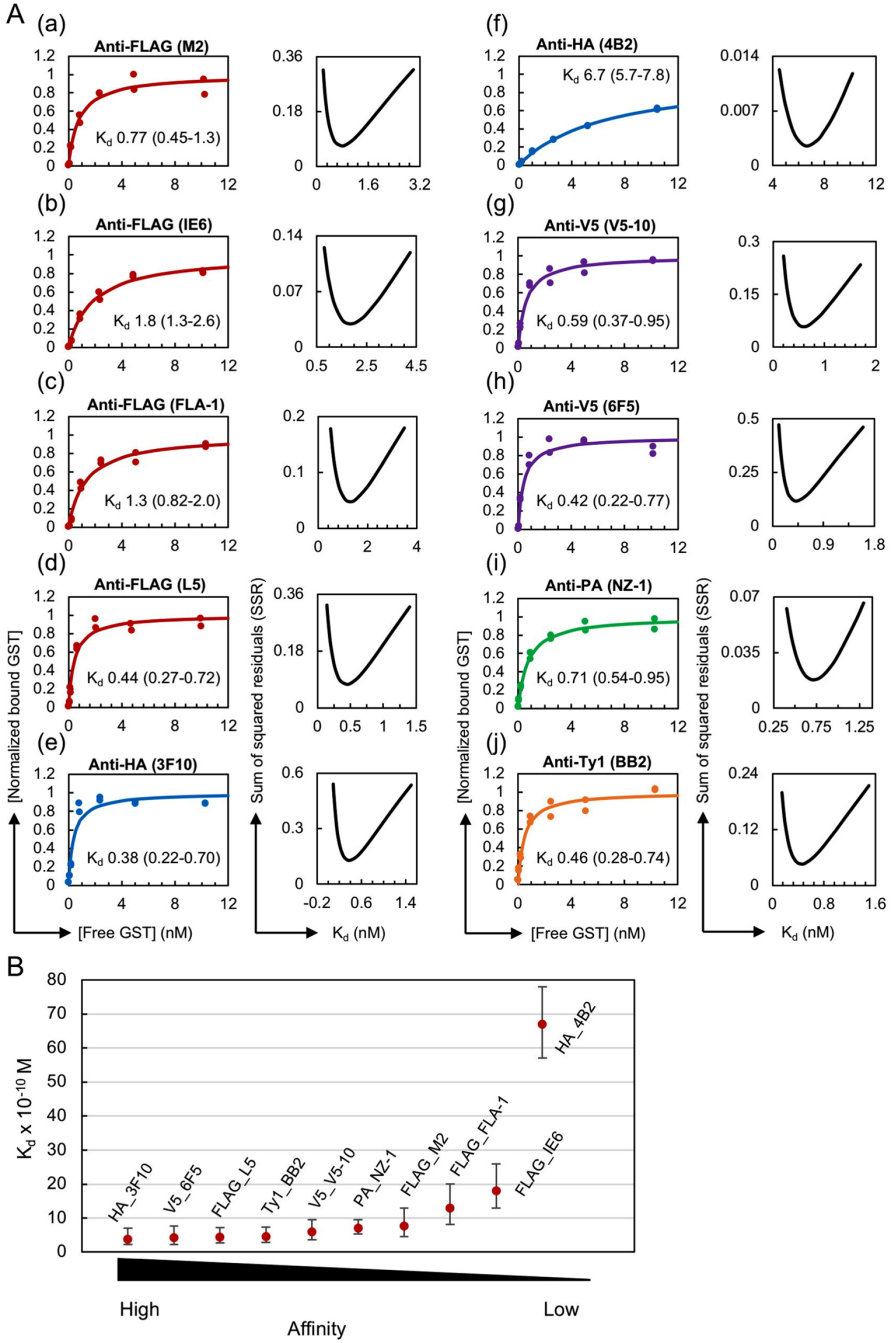
Considerable variation in K_d_ values among the epitope tag antibody clones. (**A**) (Left panel) Binding curves of the tested antibody clones against the monomeric form of the epitope tags. The antibody concentrations used for IP were as follows: 0.2 nM for anti-FLAG (M2, IE6, FLA-1 and L5) and anti-V5 (V5-10 and 6F5); 0.1 nM for anti-HA (3F10 and 4B2) and anti-PA (NZ-1); and 0.05 nM for anti-Ty1 (BB2). (Right panel) Error curves for the best-fitting K_d_. In each plot, the obtained apparent K_d_ value in nM is shown with the 95% confidence interval. (**B**) Affinity comparison of the antibody clones shown in panel A. Error bars depict the plus and minus confidence interval of the K_d_ value.

We first determined the K_d_ values of various monoclonal antibodies against the epitope tags FLAG, HA, V5, PA and Ty1, which are listed in Table 2, through the HiBiT-qIP assay using GST protein fused with their monomeric form of the tags (Fig. 2). In these assays, the epitope-tagged GST proteins at seven concentrations, ranging from 0.825 ng (~0.025 nM) to 330 ng (~10 nM), were mixed with a fixed amount of cognate monoclonal antibody such that the binding curves reached a plateau. Preliminary IP experiments revealed that anti-IgG magnetic beads more efficiently captured monoclonal antibodies, irrespective of their IgG subclasses, than protein G magnetic beads (our unpublished data, also see Kimura *et al*.^47^). Thus, the IP reactions were performed using antibodies immobilised on anti-IgG magnetic beads in 1 mL of the stringent IP buffer (known as the typical RIPA buffer), which contains 0.1% SDS, 1% Triton X-100 and 0.1% sodium deoxycholate as the detergent in Tris-buffered saline (50 mM Tris-HCl [pH 7.5], 150 mM NaCl). This IP buffer composition was selected because similar conditions have often been used in standard IP^7,40,41^ and ChIP experiments^48–50^, and ChIP is currently one of the most important applications of IP. The antibody concentration used in the IP solution was empirically adjusted and varied from 20 pM to 0.2 nM depending on the affinity of the tested antibody/antigen pair (see Materials and Methods). Each K_d_ determination experiment was conducted in duplicate, and 14 data points were used for the curve-fitting analysis (Fig. 2; the original dataset is shown in Supplementary Table 1). The error plots obtained from the K_d_ determination experiments showed a clearly defined minimum in the sum of squared residuals (SSR) (Fig. 2, right panels), validating the accuracy of the K_d_ value and the antibody concentration selected for each experiment.

A considerable variation in the K_d_ values was observed among the antibody clones examined, and these values ranged from 3.8 × 10^−10^ M for anti-HA (3F10) to 6.7 × 10^−9^ M for anti-HA (4B2) (Fig. 2A,B), but fell within a reasonable range of K_d_ values for high-affinity monoclonal antibodies, which suggests that our method exhibits high validity. The comparison of the measured K_d_ values with those available from the literature revealed both similarities and differences (Supplementary Table 2). The K_d_ value for anti-PA (NZ-1) against the dodecapeptide PA tag measured using our HiBiT-qIP assay was 7.1 × 10^−10^ M, which is close to the reported K_d_ value of 4.0 × 10^−10^ M obtained through a kinetic analysis using SPR^35^. The K_d_ value for anti-HA (4B2) obtained with our HiBiT-qIP assay was 6.3 × 10^−9^ M, which is not very different from the reported K_d_ value of 1.6 × 10^−9^ M obtained by the SPR method^35^. For anti-FLAG (M2), the obtained K_d_ value of 7.7 × 10^−10^ M deviated from those reported, which range from 3 × 10^−9^ M to 2.8 × 10^−8^ M^35,51,52^. This discrepancy might be due to differences in the position of the FLAG tag, the conditions used, including the buffer composition and pH, the method used, and/or the detection sensitivity^12,14^.

**Figure 3:**
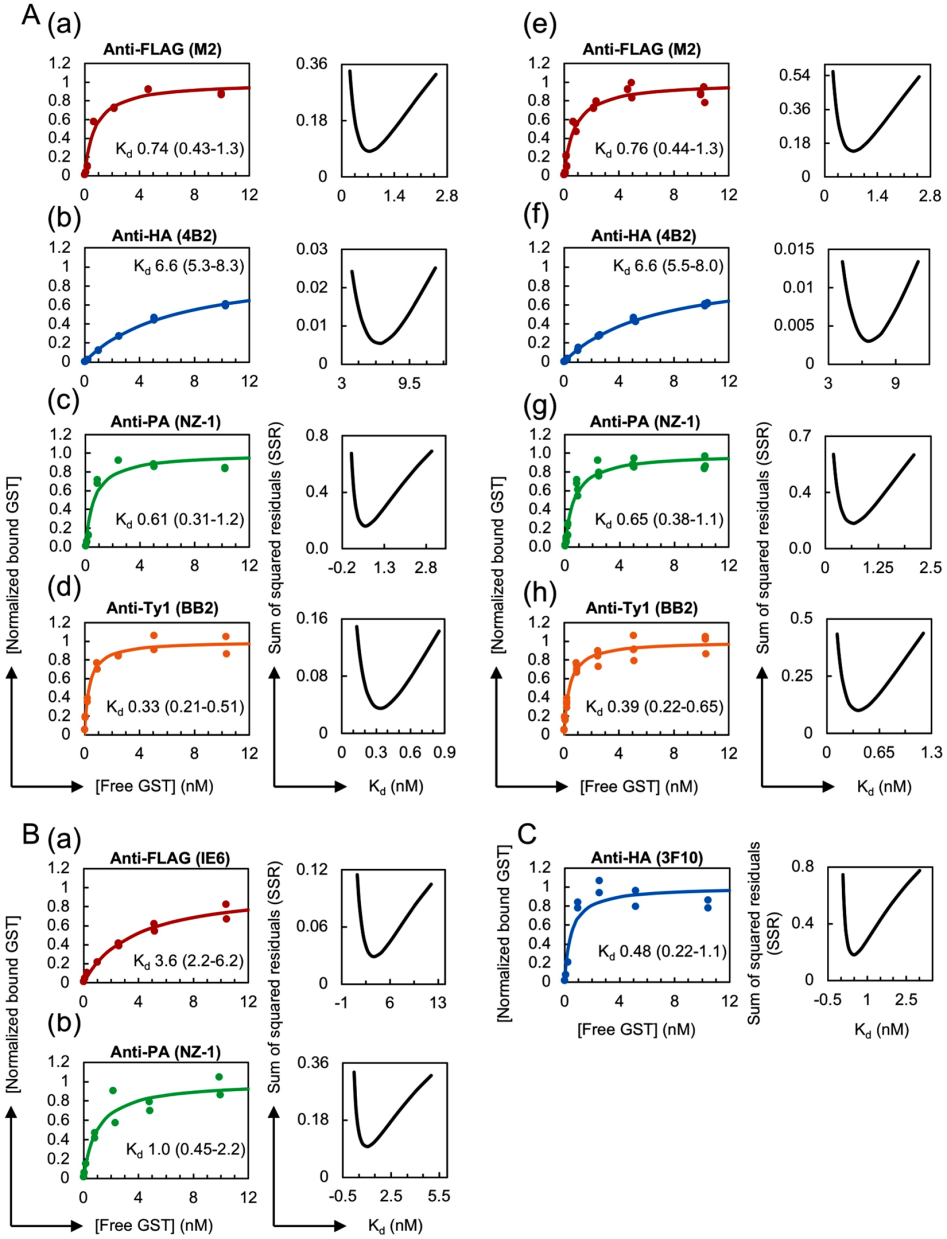
Validity and reproducibility of the HiBiT-qIP assay. (**A**) Reproducibility of the HiBiT-qIP-based K_d_ determination. (a–d) K_d_ determination experiments were repeated for four monoclonal antibody clones: anti-FLAG (M2), anti-HA (4B2), anti-PA (NZ-1) and anti-Ty1 (BB2). (Left panel) Binding curves of the antibody clones tested against the monomeric form of the epitope tags. The antibody concentrations used for IP were as follows: 0.2 nM for anti-FLAG (M2) and anti-HA (4B2); 0.1 nM for anti-PA (NZ-1); and 0.05 nM for anti-Ty1 (BB2). (e–g) Binding curves plotted with data obtained from two independent experiments, shown in Figs 2A and 3A. (**B**) IP performed using magnetic beads covalently cross-linked to anti-FLAG and anti-PA antibodies provided comparable K_d_ values. (Left panel) Binding curves of the antibody clones tested against the monomeric form of the epitope tags. The concentrations of anti-tag antibodies attached to the beads in IP were as follows: 1 nM for anti-FLAG (IE6) and 0.2 nM for anti-PA (NZ-1). (**C**) IP performed under native conditions using RIPA buffer without SDS provided a comparable K_d_ value. (Left panel) Binding curve of the anti-HA (3F10) clone against the monomeric form of HA. The concentration of the antibody used for IP was 0.1 nM. (**A**–**C**) (Right panel) Error curves for the best fit K_d_. In each plot, the obtained apparent K_d_ value is shown with the 95% confidence interval.

We repeated the measurements of the four antibody clones, anti-FLAG (M2), anti-HA (4B2), anti-PA (NZ-1) and anti-Ty1 (BB2), to assess the reproducibility of our HiBiT-qIP-based K_d_ determinations (Fig. 3Aa–d; the original dataset is shown in Supplementary Table 3) and obtained a very similar apparent K_d_ value in all cases, which indicated that the developed method shows high reproducibility. Additionally, combined data plots were generated using the data from the two independent experiments shown in Figs 2 and 3, and these plots confirmed the reproducibility of the HiBiT-qIP assay (Fig. 3Ae–h).

Notably, the rat monoclonal anti-HA (3F10), anti-FLAG (L5) and anti-PA (NZ-1) antibodies displayed significantly lower apparent K_d_ values among the clones tested, suggesting the greater utility of rat monoclonal antibodies. Among the tested anti-FLAG antibody clones, anti-FLAG (L5) exhibited a considerably lower K_d_ value than the most widely used anti-FLAG (M2), consistent with the observation that the L5 clone detects FLAG-tagged proteins more efficiently than the M2 clone in Western blotting^53^. Interestingly, the anti-Ty1 (BB2) and anti-V5 (V5-10 and 6F5) antibody clones exhibited the highest affinity among the tested mouse clones, even though the Ty1 and V5 epitope tags have been less commonly used in IP experiments than the FLAG and HA tags. This finding suggests that the Ty1 and V5 epitope tags could perform similarly to or even better than the FLAG and HA tags in IP experiments, depending on the antibody used. These results together suggest the advantage of evaluating several different clones prior to performing IP experiments and thereby identifying the most suitable clone for each epitope tag that will be used in the experiments.

In the IP procedure described above, we used the antibody-bound anti-IgG beads to capture the tagged GST proteins. Theoretically, this method measures the overall affinity of two interactions, namely, the epitope tag-antibody interaction and the antibody-anti-IgG bead interaction. In these IP reactions, however, excess amounts of anti-mouse or anti-rat IgG beads were used and most primary antibodies could be captured by the beads. Thus, it is very likely that our assay essentially measured the affinity of epitope tag-antibody interactions. To directly test this hypothesis, we used commercially available magnetic beads that have been covalently cross-linked to the anti-FLAG (IE6) mouse antibody or anti-PA (NZ-1) rat antibody. In both cases, we obtained K_d_ values that were slightly larger than those determined using the anti-IgG-bead-based protocol (Fig. 3B, Supplementary Table 3), which suggests that our assay actually measures the affinity of the epitope tag-antibody interaction.

Apparent K_d_ values could vary among different IP conditions, as noted in the Introduction. To examine these differences, if any, we performed an IP experiment using RIPA buffer without SDS because IP assays, particularly co-immunoprecipitation (Co-IP) assays, are often performed under relatively more native conditions. For this assay, the GST protein fused with a monomeric HA tag was prepared in native form and used with the anti-HA (3F10) antibody. The assay yielded a K_d_ value that was comparable to that obtained with SDS-containing RIPA buffer (Fig. 3C, Supplementary Table 3), which indicated that anti-HA (3F10) performs equally well under these two conditions.

### A significant increase in affinity was observed with the use of epitope tags in dimeric or trimeric form

**Figure 4:**
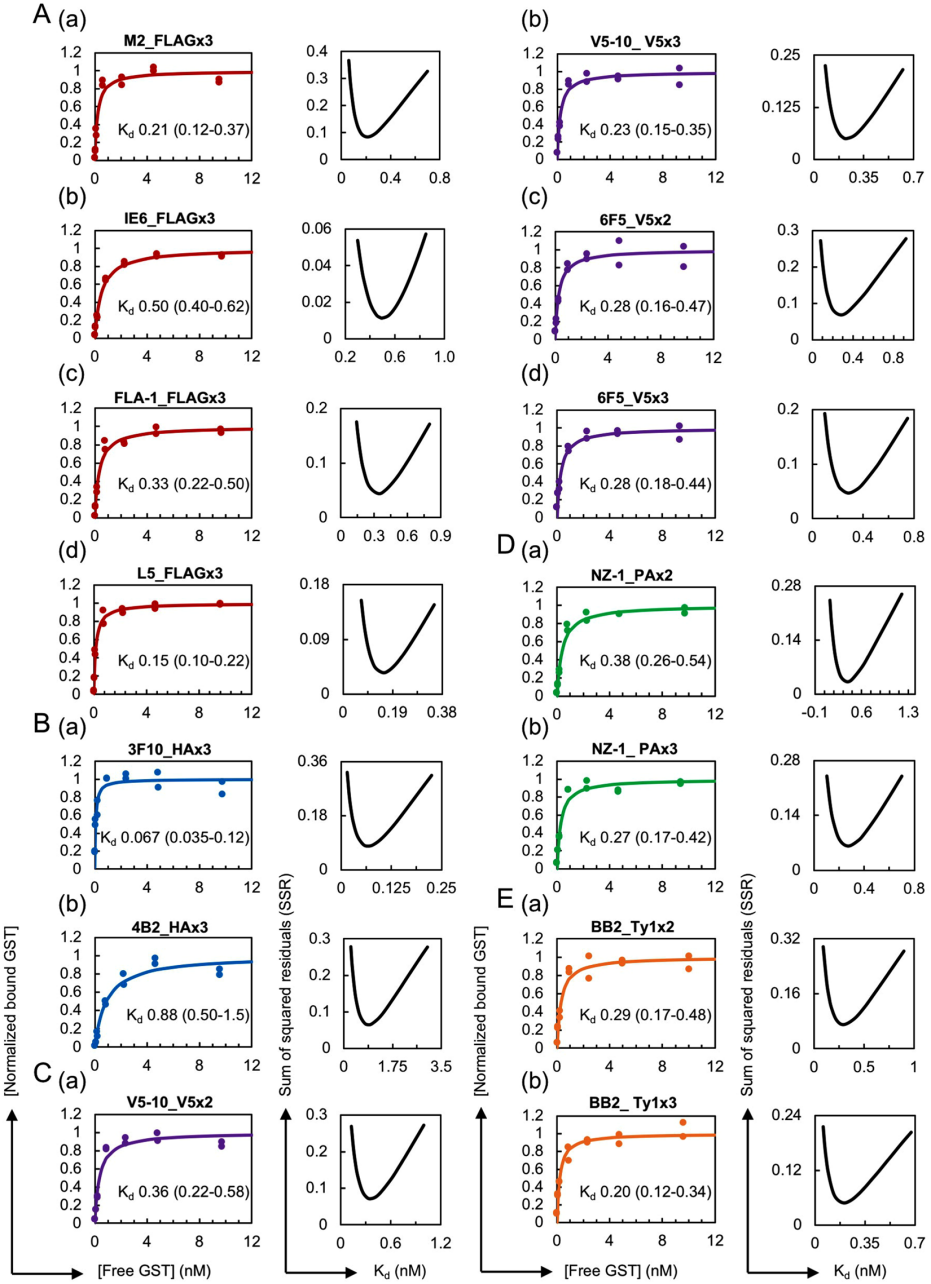
Increase in the antibody affinity against the dimeric or trimeric form of the epitope tags. (Left panel) (**A**) Binding curves of anti-FLAG M2 (a), IE6 (b), FLA-1 (c) and L5 (d) clones against FLAGx3. The antibody concentrations used for IP were 0.1 nM for (a–c) and 0.05 nM for (d). (**B**) Binding curves of the anti-HA 3F10 (a) and 4B2 (b) clones against HAx3. The antibody concentrations used for 3F10 and 4B2 were 0.02 nM and 0.1 nM, respectively. (**C**) Binding curves of the anti-V5, V5-10 and 6F5 clones against V5x2 (a,c) and V5x3 (b,d). The antibody concentration used for V5-10 was 0.05 nM for both forms. The concentrations of the antibodies used for 6F5 were 0.1 nM for V5x2 and 0.05 nM for V5x3. (**D**) Binding curves of the anti-PA NZ-1 clone against PAx2 (a) and PAx3 (b). The concentrations of the antibody used for IP were 0.1 nM for PAx2 and 0.05 nM for PAx3. (**E**) Binding curves of the anti-Ty1 BB2 clone against Ty1x2 (a) and Ty1x3 (b). The concentration of the antibody used for IP was 0.05 nM for both forms. (Right panel) Error curves for the best-fitting K_d_. In each plot, the obtained apparent K_d_ value in nM is shown with the 95% confidence interval.

**Figure 5:**
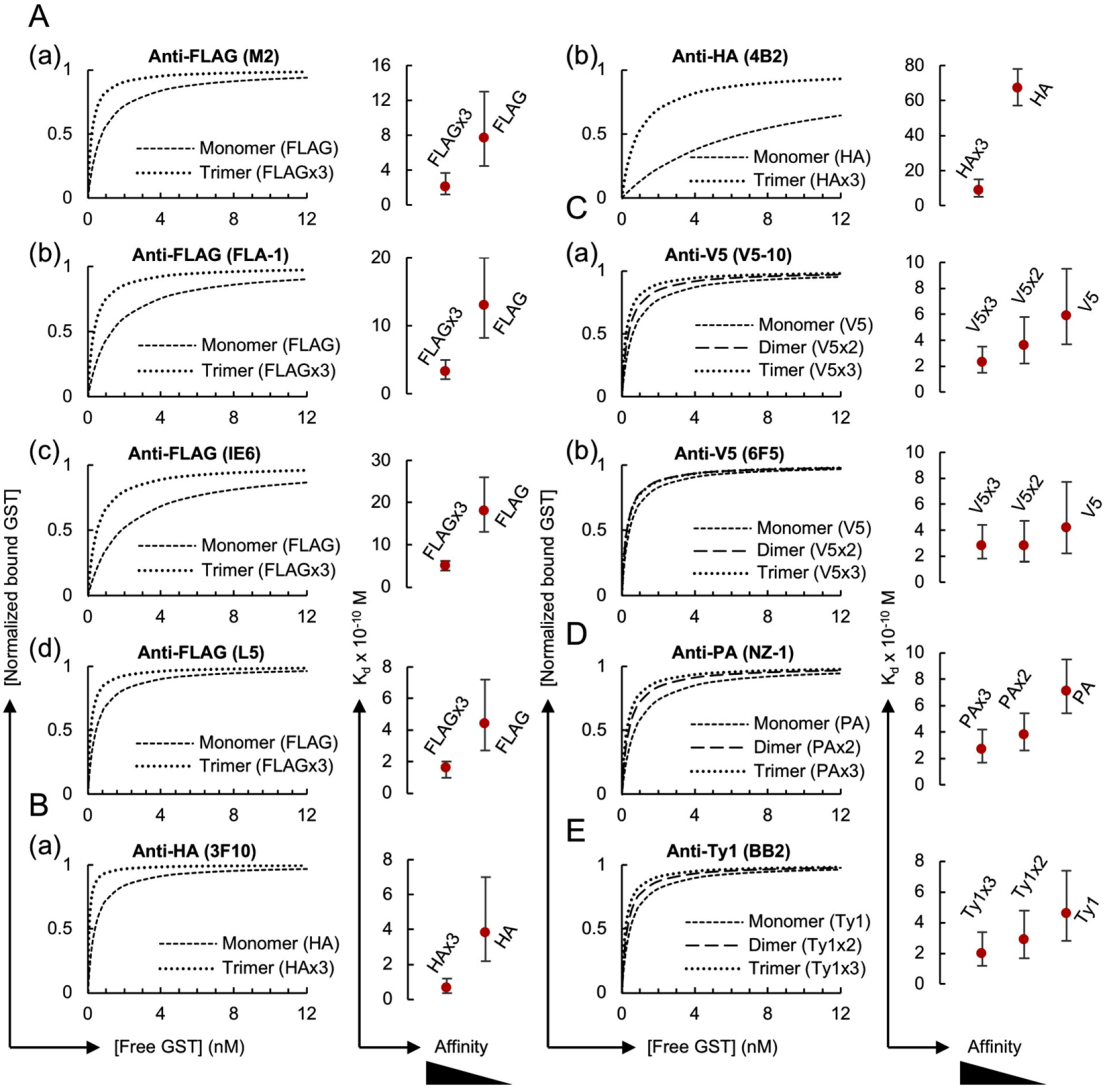
Effect of tag multimerisation on the apparent affinity of the antibodies. (Left panel) The binding curves of anti-epitope tag antibody clones against monomeric, dimeric and/or trimeric forms of the epitope tags shown Figs 2 and 4 are simultaneously plotted for comparison purposes. (Right panel) Affinity comparison. The error bars depict the 95% confidence intervals for the K_d_ values.

The dimeric or trimeric form of epitope tags has frequently been used in a variety of immunoassays to enhance their sensitivity^38,54–56^, but the effects of multimerisation in immunoprecipitation have not yet been quantitatively characterised. To address this problem, we measured the apparent K_d_ values for the dimeric and trimeric forms of the epitope tags based on the assumption that a one-to-one interaction primarily occurs between the antibody and the multimerised epitope tag peptide under our assay conditions (see Discussion). Here, we thus use the term “apparent K_d_” as an artificial parameter to describe the interaction between the antibody and the multimerised tag by considering the multimerised tag as a single binding site. We produced GST proteins with trimeric forms of FLAG and HA and GST proteins with dimeric and trimeric forms of V5, PA and Ty1 (Table 1, Supplementary Fig. 1). Our trimeric form of FLAG consisted of simple direct repeats of DYKDDDDK, and was thus not identical to the original 3xFLAG sequence DYKDHDGDYKDHDIDYKDDDDK, in which modified FLAG sequences are used in the first and second positions^56^. We selected this simple repeated form because the original 3xFLAG was optimised for the traditional anti-FLAG (M2) clone and might thus not be recognised by the newly developed anti-FLAG monoclonal antibodies used in this study. The use of these epitope-tagged GST proteins in the HiBiT-qIP assay revealed a several-fold increase in the apparent affinity compared with that obtained with the monomeric forms (Figs 4 and 5, Table 2; the original data set is shown in Supplementary Table 4). The comparison of the mono-, di- and trimeric forms showed a gradual increase in affinity depending on the number of epitope tags (Fig. 5C–E), indicating a clear positive correlation between the apparent affinity and the number of epitopes; however, the differences in affinity between the dimeric and trimeric forms were rather small, particularly those of anti-V5 (6F5) (Fig. 5Cb).

A comparison of the mono- and trimeric forms of FLAG and HA revealed a considerable increase in the affinity of all the tested antibodies with the use of their trimeric forms. The anti-HA 3F10 clone with the lowest K_d_ value in our comparisons exhibited increased affinity against the trimeric form of HA, and showed the highest affinity among the clones tested (Figs 2B, 4Ba and 5Ba). Interestingly, the difference in apparent affinities among the four anti-FLAG monoclonal clones decreased when examined against the trimeric form of the FLAG tag. This phenomenon was also clearly observed with anti-HA (4B2). Taken together, these results indicate that the dimerisation and trimerisation of epitope tags clearly increase the apparent affinity of antibodies under IP conditions. In addition, the results suggest that in cases in which high-affinity antibodies are unavailable, low-affinity antibodies might be successfully used in IP experiments if combined with multimeric forms of the epitope tags.

### Tag multimerisation greatly improved the efficiency of IP from crude cell lysates

**Figure 6:**
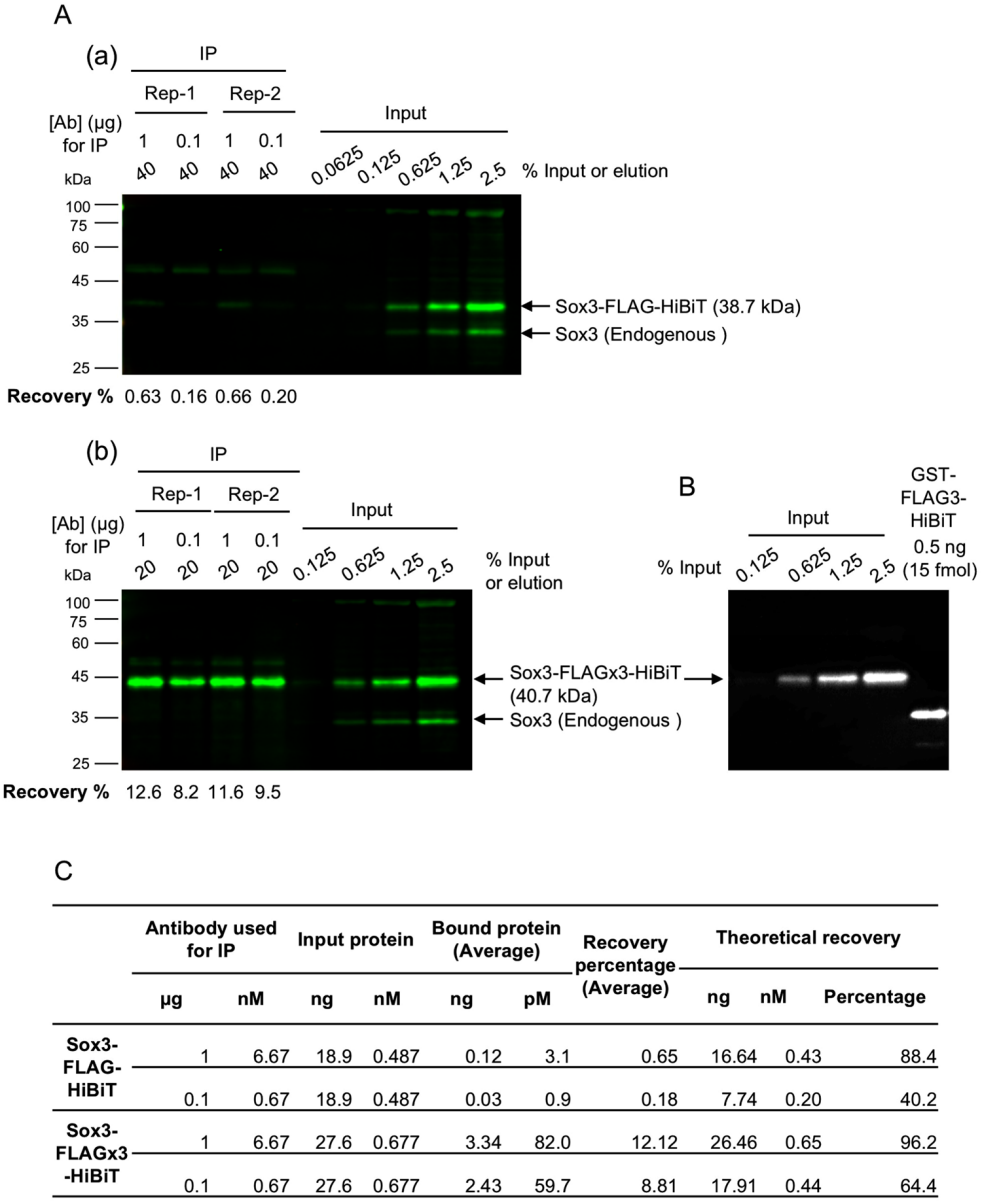
Effect of tag multimerisation on the efficiency of IP from crude cell lysates. (**A**) Infrared fluorescent Western blot detection of the FLAG-tagged Sox3 proteins. The Sox3 protein tagged with FLAG either in monomeric (a) or trimeric (b) form in IP samples and the dilution series of input lysates were detected using anti-Sox3 antibody. IP was performed in duplicate and under two IP conditions, i.e., 1 µg or 0.1 µg of anti-FLAG (IE6) antibody was incubated with the embryo lysates in 1 mL of IP solution. The recovery rates (in percentages) of FLAG-tagged Sox3 are shown at the bottom of each blot. The two blots in panels (a,b) were processed in parallel and scanned simultaneously. (**B**) HiBiT detection of input dilutions of FLAGx3-tagged Sox3 on the same membrane shown in (Ab) and 0.5 ng (15 fmol) of FLAGx3-tagged GST, which was used as the standard for absolute protein quantification. (**C**) Comparison of the experimental and theoretical IP recovery values. The averaged recovery rates of duplicated IP experiments and theoretical recovery rates are shown. The process used for the theoretical recovery calculation is given in the Materials and Methods section.

Because a significant increase in affinity was observed with the use of epitope tags in multimeric form, we questioned the resulting effects on the efficiency of IP from crude cell lysates, which is closer to real experimental conditions. To answer this question, we synthesised mRNAs encoding the zebrafish transcription factor Sox3 tagged with a monomeric or trimeric form of the FLAG tag and HiBiT, expressed these proteins in zebrafish embryos at near-endogenous levels, and prepared crude cell lysates in RIPA buffer containing SDS. We immunoprecipitated FLAG-tagged Sox3 using an anti-FLAG (IE6) antibody and quantified the amount of recovered Sox3 proteins. Specifically, Western blotting with an anti-Sox3 antibody was used to determine the relative amounts of Sox3, and HiBiT blotting using GST-FLAGx3-HiBiT as a standard was performed to convert the relative amounts to absolute amounts (Fig. 6, Supplementary Fig. 3). In accordance with the differences in affinity, a considerable improvement in IP recovery was clearly observed with the use of the trimeric form of the FLAG tag (Fig. 6A,C), and this effect was more pronounced if a limited amount of antibody was used. Interestingly, the comparison of these observed recovery rates with those calculated theoretically based on the K_d_ value revealed a substantial difference, particularly for the monomeric FLAG tag (Fig. 6C, see Discussion).

## Discussion

### Determination of K_d_ values through the HiBiT-qIP assay

In the current study, we employed the NanoLuc-based HiBiT system^20,21^ to establish our HiBiT-qIP assay for determining the K_d_ values for protein-protein interactions in solution. We applied this method to measure the K_d_ values for interactions between certain epitope tags and a series of their cognate monoclonal antibodies under the IP conditions generally used in ChIP. We assumed that the antigen-antibody interaction reaches equilibrium during the overnight IP and that the steady-state dissociation constant could be determined by measuring the amount of immunoprecipitated proteins after a brief wash. Due to the high sensitivity of the HiBiT solution assay, in which a sub-picogram protein amount can be detected within the linear response region, we were able to perform titration experiments with a wide range of antigen concentrations. In fact, the apparent K_d_ values for the tested monoclonal antibody clones were found to be between 10^−9^ M and 10^−11^ M, which is the typical range of the reported K_d_ values for high-affinity antigen-antibody interactions^7,45^. Furthermore, for the anti-PA antibody, we obtained a K_d_ value similar to that reported^35^. To evaluate the accuracy of the K_d_ values obtained and the antibody concentration selected for each experiment, the 95% confidence intervals (CIs) were assessed as previously described^57,58^. In most cases, we were able to obtain K_d_ values with 95% confidence intervals from half of the K_d_ to twice the K_d_. These findings strongly suggest that the HiBiT-qIP assay is able to measure K_d_ values with reasonably good accuracy.

Notably, although we used the HiBiT-qIP assay to measure the K_d_ values for anti-epitope tag monoclonal antibodies, this method could be used to obtain the K_d_ values for any type of monoclonal antibody as long as the target protein can be tagged with HiBiT. Moreover, in theory, the K_d_ values for any type of protein-protein interaction in solution might be obtained if one can prepare an appropriate combination of a HiBiT-tagged protein and another protein that can be captured with beads through covalent crosslinking or a high affinity interaction such as avidin-biotin.

Only a few methods have been developed for quantitatively characterising antigen-antibody interactions under IP conditions, which has resulted in difficulties in selecting suitable antibodies for demanding IP applications such as ChIP^2,6^. To overcome this problem, a quantitative peptide immunoprecipitation (peptide IP) assay in a ChIP-like format was developed by Nishikori *et al*.^59^. In their method, a biotinylated antigen peptide is incubated with antibody-bound protein A (or G) polystyrene beads in solution. The captured peptide is then linked to fluorescently labelled streptavidin and quantified using flow cytometry. The advantage of their assay is that it is readily applicable if a biotinylated antigen peptide is available, but an inherent drawback is that the antigen-antibody complex might dissociate during flow cytometry and the IP wash process, which could lead to underestimation of the antibody affinity. Our HiBiT-qIP assay has the same dissociation problem during the IP wash process, but the effect can be minimised by performing the wash process reasonably rapidly. Our HiBiT-qIP assay and the peptide IP assay developed by Nishikori *et al*.^59^ technically measure the overall affinity of all the interactions involved in the process: the former assay measures the overall affinity of the antibody-antigen and antibody-bead interactions, whereas the latter measures the overall affinity of the antibody-peptide, antibody-bead and biotin-streptavidin interactions. However, as discussed by Hattori *et al*.^60^, these IP-based assays appear to essentially measure the affinity of the antibody-antigen interactions because most of the primary antibodies are expected to be captured by the capture beads, which are added in excess amounts in IP reactions. Consistent with this notion, we obtained similar apparent K_d_ values even with magnetic beads that had been covalently cross-linked to tag antibodies (Fig. 3B). However, this finding also implies that it might be difficult to measure interactions with affinities higher than those of the antibody-bead interaction in these standard IP-based assays.

At present, the most widely used methods for determining the K_d_ values of an antigen-antibody interactions are SPR technology^10,11^ and KinExA^12,13^. KinExA allows the direct measurement of the equilibrium binding affinity of interactions between molecules in solution after an equilibrium is reached. In contrast, in the SPR approach, the kinetic parameters, the association rate constant (k_on_) and the dissociation rate constant (k_off_) are first determined, and these parameters are then used to calculate K_d_ as K_d_ = k_off_/k_on_. Due to methodological similarities, a K_d_ value obtained using the HiBiT-qIP assay might be closer to that measured by KinExA. Because SPR technology might not accurately measure very slow k_off_ values in a standard flow mode, it might be difficult to measure high-affinity interactions with low k_off_ values using this method^61^. In contrast, the HiBiT-qIP assay cannot be applied to measure interactions with high k_off_ values because this method involves a wash process after IP, as discussed above. The advantages of the HiBiT-qIP assay compared with SPR and KinExA are summarised as follows: (1) this method can directly measure apparent K_d_ values under specific IP buffer conditions, (2) this method can be performed with small amounts of antibody and antigen, and (3) this method only uses a standard luminometer and thus provides a more accessible approach for determining K_d_ values.

### Evaluation of epitope tag antibodies

It has been suggested that immunoprecipitation experiments normally require antibody affinities of at least 10^−8^ M for efficient recovery because they rely on the formation of an antigen-antibody complex in solution at relatively low concentrations of the antigen^7^. The monoclonal anti-epitope tag antibodies tested in this study all meet this criterion, which is consistent with the fact that they are supplied as antibodies that can be used for IP. For more demanding IP experiments, however, higher-affinity antibodies with K_d_ values equal to or less than 10^−9^ M might be required.

Our results clearly show a several-fold increase in the apparent affinity by the use of any of the epitope tags in dimeric or trimeric form. This effect can be explained in part by the fact that the use of multimeric tags leads to a simple increase in molar concentration of each monomeric tag. Additionally, the close proximity of the epitopes on the multimeric tags might facilitate rebinding of the antibody to the neighbouring sites. On the other hand, bivalent binding of an antibody to a single multimerised tag may not be possible due to a large distance between the two antigen binding sites relative to the length of tag polypeptides^62^. Moreover, simultaneous binding of a multimeric tag to neighbouring antibodies on a magnetic bead also seems to be a rare case because surface density of the antibodies on the bead is calculated to be low (less than one antibody/2300 nm^2^) under our IP conditions. Historically, Hernan *et al*.^56^ discovered that the Western blot detection limit can be improved by more than 10-fold by tagging a target protein with a sequence containing two additional FLAG epitopes in tandem with the original FLAG sequence (3x FLAG). Since then, the 3x FLAG epitope tag has been widely used due to its enhanced sensitivity in affinity isolation and immunohistochemical detection. Remarkably, epitope tagging with the triple-FLAG tag facilitated the IP of low-abundance proteins at near-endogenous levels, whereas the FLAG monomer failed to immunoprecipitate the proteins^55^. Consistent with this, we observed a considerable improvement in IP recovery with the use of the trimeric FLAG tag (Fig. 6). This enhancing effect can be clearly observed in IP experiments performed with limited amounts of the target protein. In fact, Zhang *et al*.^63^ observed equal precipitation of both the monomeric and trimeric forms of FLAG-tagged target proteins in their IP experiments. Consistent with this, under our IP conditions, we clearly observed a substantial increase in the HiBiT-derived signal from the immunoprecipitate of the FLAG trimer compared with that obtained with the FLAG monomer only if lower amounts of FLAG-tagged GST were used (Fig. 5). The IP of FLAG-tagged Sox3 from the crude cell lysates with the monomeric FLAG tag exhibited a substantially lower IP recovery compared with that obtained with the trimeric FLAG tag. This difference cannot be explained by the affinity difference. However, it is possible that some protein components in the crude lysate might strongly inhibit the antibody-tag interaction and this inhibition might have been overcome by tag multimerisation. Interestingly, our results show that not only the FLAG tag but also the rest of the epitope tags we tested exhibited improved affinity and therefore an increased IP yield when used in multiple tandem repeats. This finding reflects the wide use of epitope tags in their multimerised forms, although the biochemical basis has been rarely examined.

The utility of an antibody in IP is critically dependent on its specificity in addition to its affinity, although we did not address this point in this study. A recent study by Macron *et al*.^64^ showed that antibody selectivity and specificity in IP can be effectively characterised by quantifying the abundance of all the proteins in the immunoprecipitates. This approach is complementary to that presented in this paper, and these two approaches can support each other. Thus, high affinity is not the sole criterion for selecting good antibodies for IP experiments but might be the most important factor because a high-affinity interaction enables the performance of IP experiments under stringent conditions, which would result in an increase in specificity. Overall, this comparison of monoclonal antibodies against the commonly used epitope tags will prove to be a valuable resource for future IP-related experiments.

Finally, we suggest that our HiBiT-qIP assay might also be useful in quantitative monitoring of IP experiments. As mentioned in the Introduction and elaborated in Hakhverdyan *et al*.^65^, the efficiency of IP is strongly influenced by reagents used in IP solution such as salts, buffers and detergents. The efficiency of IP is also affected by the complexity of protein samples, as observed in this study (Fig. 6). However, it is unpredictable how these factors affect the performance of IP^65^. Thus, it would be necessary to explore the parameters affecting the efficiency of IP, particularly when the target protein is expressed at near-endogenous levels. Under such circumstances, HiBiT-qIP could facilitate the evaluation of various IP parameters through quantitative analysis of the immunoprecipitated proteins tagged with HiBiT.

## Materials and Methods

### Plasmid DNA construction for epitope-tagged GST protein expression

Using pGEX-6P-1 (GE Healthcare) as the parental vector, the coding region of the glutathione S-transferase (GST) gene was fused in-frame to a series of composite tags at the *Xho*I site in the multi-cloning sites. Each of the composite tags contained one of the epitope tags, namely, FLAG, HA, PA, V5 and Ty1 in either monomeric, dimeric or trimeric form, followed by a TEV protease cleavage site, a biotin acceptor domain (Bio tag) and, most C-terminally, the HiBiT epitope tag. The exact nucleotide and amino acid sequences of the composite epitope tags are listed in Supplementary Fig. 1.

### Epitope tag antibodies

The monoclonal antibodies used for immunoprecipitation are provided, along with suppliers, clone IDs, host species, IgG subclasses and the type of magnetic beads, in Table 2. The product numbers of these antibodies are as follows: anti-FLAG (M2), F1804; anti-FLAG (FLA-1), M185-3S; anti-FLAG (IE6), 018-22386; anti-FLAG (L5), 637301; anti-HA (3F10), 11867423001; anti-HA (4B2), 010-21883; anti-PA (NZ-1), 016-25861; anti-V5 (V5-10), V8012; anti-V5 (6F5), 017-23593; and anti-Ty1 (BB2), SAB4800032.

### Expression and purification of the epitope-tagged GST proteins

The GEX-6P-1-derived plasmids containing the various composite tag sequences were transformed into *Escherichia coli* (JM109) cells for protein expression. A single colony of transformed *E. coli* cells was inoculated in 2xYT medium and incubated overnight at 37 °C with vigorous shaking. The culture was diluted 1:100 into 5 mL of fresh 2xYT medium and incubated at 28 °C with shaking until the A_600_ reached 0.6–0.8. Protein expression was induced by the addition of IPTG (0.1 mM), and the cells were then incubated for an additional 3 hours, pelleted by centrifugation, resuspended in TBS (Tris-buffered saline: 20 mM Tris-HCl [pH 7.5] and 150 mM NaCl) and lysed by sonication (Bioruptor, Cosmo Bio) until the suspension became clear. The soluble fraction, which was separated from the insoluble fraction by centrifugation (15,000 × g, 4 °C, 5 min), was mixed with glutathione–Sepharose beads (GE Healthcare) and rotated for 10 min at room temperature (RT). The resin was collected by brief centrifugation, and unbound proteins were washed away from the beads with TBS. The GST fusion proteins were then eluted by the addition of nuclei lysis buffer (50 mM Tris-HCl [pH 7.5], and10 mM EDTA, 1% [w/v] SDS). In addition, to elute the GST proteins in native form, glutathione elution buffer consisting of 10 mM reduced glutathione and 50 mM Tris-HCl (pH 8.0) was used. The purified proteins were subsequently separated using 10% SDS-polyacrylamide gels, stained with Coomassie Brilliant Blue G-250 and scanned with an Odyssey instrument (LI-COR) using the 700-nm channel^39^. The scanned image was used to quantify the protein amounts using BSA as the standard.

### Nano-Glo HiBiT blotting

To detect the HiBiT-derived signal from the GST proteins fused with monomeric, dimeric and trimeric forms of the epitope tags and the HiBiT peptide, equal amounts of the purified proteins were separated on 10% SDS-polyacrylamide gels and transferred to nitrocellulose membranes. The protein-transferred membranes were incubated in TBST for 30 min, and this medium was then replaced with Nano-Glo HiBiT blotting reagent containing LgBiT protein (Promega). After 1 hour of incubation at RT, the substrate furimazine was added, and the incubation was continued for another 5 min. The blot was imaged using a chemiluminescence imager with a CCD camera (Fusion, Vilber Lourmat).

### Immunoprecipitation

Varying concentrations of the purified epitope-tagged GST proteins were prepared in nuclei lysis buffer and diluted 10-fold with ChIP dilution buffer (50 mM Tris-HCl [pH 7.5], 167 mM NaCl, 1.1% Triton X-100, and 0.11% [w/v] sodium deoxycholate) to obtain the RIPA buffer composition (50 mM Tris-HCl [pH 7.5], 150 mM NaCl, 1.0% Triton X-100, 0.1% sodium deoxycholate, and 0.1% SDS). One millilitre of the diluted protein sample was mixed with 15 µL of anti-mouse or anti-rat IgG magnetic beads (~10 mg/mL, Invitrogen) in PBS supplemented with 0.5% BSA (Rockland Immunochemicals) that had been pre-bound to 30 ng (or lower, i.e., 15 ng, 7.5 ng or 3 ng, as stated in the figure legends) of the anti-epitope tag antibody. The beads were incubated overnight at 4 °C and then washed twice with ice-cold HEPES-RIPA buffer (50 mM HEPES [pH 7.6], 1 mM EDTA, 0.7% [w/v] sodium deoxycholate, 1% Triton X-100, and 0.5 M LiCl) and once with ice-cold TBS buffer. Any residual TBS was removed by centrifugation followed by aspiration. The immunoprecipitated protein was then eluted with 20 µL of elution buffer containing 0.1% (w/v) SDS, 50 mM Tris-HCl, 10 mM EDTA and 25 mM DDT for 5 min at 95 °C. The supernatant was collected through a brief centrifugation (18,800 × g, 1 min). For each K_d_ determination, a fixed amount of antibody was used, while the epitope-tagged GST proteins were titrated as a dilution series ranging from 0.825 ng/mL (~0.025 nM) to 330 ng/mL (~10 nM) to obtain seven data points. Each point was analysed in duplicate independent samples to ensure the determination of an accurate K_d_. The amount of antibody used was empirically optimised based on preliminary experiments such that the antibody concentration was maintained at a level lower than the K_d_ value. Specifically, the antibody amount that maintained at least half of the epitope-tagged GST protein in the unbound state, particularly for the data point corresponding to the lowest GST concentration, was considered the best suitable antibody concentration for IP. Given that the molecular mass of IgG is 150 kDa, 30 ng, 15 ng, 7.5 ng and 3 ng of an antibody in 1 mL of immunoprecipitation reaction buffer correspond to concentrations of 0.2 nM, 0.1 nM, 0.05 nM and 0.02 nM, respectively. In the experiment involving magnetic beads covalently cross-linked to tag antibodies, we used anti-DYKDDDDK tag antibody magnetic beads (clone IE6, Wako) and MagCapture HP anti-PA tag antibody magnetic beads (clone NZ-1, Wako).

### HiBiT detection assays

The immunoprecipitated samples were diluted 100-fold using PBS containing 0.01% BSA and 0.1% Triton X-100, and 20 µL of these diluted samples was mixed with an equal volume of Nano-Glo HiBiT Lytic Reagent (Promega), consisting of Nano-Glo HiBiT Lytic Buffer, Nano-Glo HiBiT Lytic Substrate and LgBiT protein. This mixture was incubated for 10 min at RT, and the luminescence was measured using a Mithras LB940 plate reader (Berthold Technologies) with an integration time of 1 s. The amounts of the HiBiT tag were calculated using the same epitope-tagged GST protein as the standard.

### Determination of apparent K_d_

The overnight incubation of IP samples at 4 °C is expected to allow the binding reaction between the antigen and antibody to reach equilibrium. The bound epitope-tagged GST proteins were eluted, and the amount was determined using the HiBiT detection assays as described above. The apparent K_d_ was determined by fitting the data to the following equation^66^:

$$[{{\rm{L}}}_{{\rm{b}}}]/[{{\rm{L}}}_{{\rm{b}}\_{\rm{\max }}}]=[{{\rm{L}}}_{{\rm{f}}}]/({{\rm{K}}}_{{\rm{d}}}+[{{\rm{L}}}_{{\rm{f}}}]),$$

where [L_b_] is the bound GST concentration (observed), [L_b_max_] is the maximum bound GST concentration (calculated), and [L_f_] is the free GST concentration ([L_total_] − [L_b_]).

Nonlinear least-squares data fitting was accomplished using the Solver add-in regression tool built in Microsoft Excel. Here, we first obtained the value of [L_b_max_], and using these values, we then re-plotted the data to draw the final fitted curves that are shown in the figures, in which [L_b_]/[L_b_max_] is the normalised bound GST value. To assess the best-fit parameter values returned by the nonlinear regression, a 95% confidence interval was calculated using Fisher’s F distribution, as elaborated by Kemmer *et al*.^57^. The step-by-step procedure is also shown in the first sheet of Supplementary Table 1.

### mRNA synthesis and zebrafish embryo microinjection

To construct plasmids for the synthesis of mRNA encoding the zebrafish Sox3 protein tagged with a monomeric or trimeric form of the FLAG tag and the HiBiT tag, we inserted the zebrafish *sox3* coding sequence and the composite epitope tags into pCS2. The capped mRNAs for these FLAG-tagged Sox3 proteins were transcribed *in vitro* from linearised vectors using the mMessage mMachine SP6 kit (Ambion, ThermoFisher). Zebrafish embryos were obtained from the natural mating of wild-type TL fish and reared at 28.5 °C in 0.03% Red Sea salt solution. Approximately 1 nL of solution containing FLAG-tagged Sox3 mRNA at a concentration of 10 ng/µL was injected into one -cell-stage embryos. The mRNA encoding the Venus fluorescent protein was included in the injection solution at a concentration of 50 ng/µL and used as a reporter to confirm the success of the microinjection. All zebrafish experiments were conducted in accordance with the Fundamental Guidelines for Proper Conduct of Animal Experiment and Related Activities in Academic Research Institutions under the jurisdiction of the Ministry of Education, Culture, Sports, Science and Technology of Japan using protocols approved by the Animal Experiments Committee of Osaka University. The same protocols were used at Kochi University of Technology.

### IP followed by Western blotting using zebrafish embryo lysates

Microinjected zebrafish embryos at the 70–80% epiboly stage were enzymatically dechorionated with Pronase (2 mg/mL) and deyolked as described by Link *et al*.^67^ One embryo/µL of embryo sample was prepared in nuclei lysis buffer containing the complete protease inhibitor cocktail (Roche). For each IP experiment, an embryo lysate equivalent to 100 embryos was sonicated and diluted 10-fold with ChIP dilution buffer containing protease inhibitors. IP was performed as described above, and proteins were eluted in 100 µL of SDS sample buffer containing 50 mM DTT. The samples were denatured by exposure to heat at 95 °C for 10 min, and after a brief centrifugation (18,800 × g, 1 min), the supernatant was collected. The immunoprecipitated samples were then separated by SDS-PAGE along with a dilution series of input samples and transferred to nitrocellulose membranes (BioTrace NT, Pall Corporation) using a TransBlot Cell (Bio-Rad). The membrane was then blocked with Odyssey blocking buffer (1:1 diluted with TBS), rinsed with TBST buffer (TBS and 0.05% Tween-20), and probed with anti-Sox3 (GTX132494, GeneTex) at 0.5 µg/mL diluted in Can Get Signal solution 1 (Toyobo). The Sox-3 antibody was detected with goat anti-rabbit IgG-IRDye800 using an Odyssey CLx infrared imaging system (LI-COR Biosciences). The band intensities of Western blot images were quantified using Image Studio software (LI-COR Biosciences).

### Theoretical IP recovery

The K_d_ values obtained for the anti-FLAG (IE6) against the monomeric and trimeric forms of FLAG tag were used to calculate the theoretical IP recovery rates using the following equation:

$${\rm{b}}=[({{\rm{K}}}_{{\rm{d}}}+{\rm{L}}+{\rm{R}})-\surd {({{\rm{K}}}_{{\rm{d}}}+{\rm{L}}+{\rm{R}})}^{2}-4{\rm{LR}}]/2,$$

where b is the concentration of bound antibody, L is the total concentration of antigen, and R is the total concentration of binding sites on the antibody^66^.

## Supplementary information

Supplementary Information

Supplementary Table 1

Supplementary Table 3

Supplementary Table 4

## References

[1] Bordeaux J (2010). Antibody validation. Biotechniques.

[2] Egelhofer TA (2011). An assessment of histone-modification antibody quality. Nat. Struct. Mol. Biol..

[3] Weller MG (2018). Ten basic rules of antibody validation. Anal. Chem. Insights.

[4] Reiss PD, Min D, Leung MY (2014). Working towards a consensus for antibody validation. F1000Research.

[5] Acharya P, Quinlan A, Neumeister V (2017). The ABCs of finding a good antibody: How to find a good antibody, validate it, and publish meaningful data. F1000Research.

[6] Wardle FC, Tan H (2015). A ChIP on the shoulder? Chromatin immunoprecipitation and validation strategies for ChIP antibodies. F1000Research.

[7] Greenfield, E. A. *Antibodies: A laboratory manual. Second edition*. (Cold Spring Harbor Laboratory Press, 2014).

[8] Kidder BL, Hu G, Zhao K (2011). ChIP-Seq: technical considerations for obtaining high-quality data. Nat. Immunol..

[9] Friguet B, Chaffotte AF, Djavadi-Ohaniance L, Goldberg ME (1985). Measurements of the true affinity constant in solution of antigen-antibody complexes by enzyme-linked immunosorbent assay. J. Immunol. Methods.

[10] Myszka DG (2000). Kinetic, equilibrium, and thermodynamic analysis of macromolecular interactions with BIACORE in. Methods in Enzymology.

[11] Neri D, Montigiani S, Kirkham PM (1996). Biophysical methods for the determination of antibody-antigen affinities. Trends Biotechnol..

[12] Glass TR, Ohmura N, Saiki H (2007). Least detectable concentration and dynamic range of three immunoassay systems using the same antibody. Anal. Chem..

[13] Bee C (2012). Exploring the dynamic range of the kinetic exclusion assay in characterizing antigen-antibody interactions. PLoS One.

[14] Drake AW (2012). Biacore surface matrix effects on the binding kinetics and affinity of an antigen/antibody complex. Anal. Biochem..

[15] Heinrich L, Tissot N, Hartmann DJ, Cohen R (2010). Comparison of the results obtained by ELISA and surface plasmon resonance for the determination of antibody affinity. J. Immunol. Methods.

[16] Reverberi R, Reverberi L (2007). Factors affecting the antigen-antibody reaction. Blood Transfus..

[17] Dimitriadis GJ (1979). Effect of detergents on antibody-antigen interaction. Anal. Biochem..

[18] Wang YV (2007). Quantitative analyses reveal the importance of regulated Hdmx degradation for P53 activation. Proc. Natl. Acad. Sci. USA.

[19] Janes KA (2015). An analysis of critical factors for quantitative immunoblotting. Sci. Signal..

[20] Dixon AS (2016). NanoLuc complementation reporter optimized for accurate measurement of protein interactions in cells. ACS Chem. Biol..

[21] Schwinn MK (2018). CRISPR-mediated tagging of endogenous proteins with a luminescent peptide. ACS Chem. Biol..

[22] Oh-hashi K, Furuta E, Fujimura K, Hirata Y (2017). Application of a novel HiBiT peptide tag for monitoring ATF4 protein expression in Neuro2a cells. Biochem. Biophys. Reports.

[23] Sasaki M (2018). Development of a rapid and quantitative method for the analysis of viral entry and release using a NanoLuc luciferase complementation assay. Virus Res..

[24] Brizzard B (2008). Epitope tagging. Biotechniques.

[25] Maue RA (2007). Understanding ion channel biology using epitope tags: Progress, pitfalls, and promise. J. Cell. Physiol..

[26] Kanca O, Bellen HJ, Schnorrer F (2017). Gene tagging strategies to assess protein expression, localization, and function in drosophila. Genetics.

[27] Partridge EC, Watkins TA, Mendenhall EM (2016). Every transcription factor deserves its map: Scaling up epitope tagging of proteins to bypass antibody problems. BioEssays.

[28] Savic D (2015). CETCh-seq: CRISPR epitope tagging ChIP-seq of DNA-binding proteins. Genome Res..

[29] Yang H (2013). One-step generation of mice carrying reporter and conditional alleles by CRISPR/Cas-mediated genome engineering. Cell.

[30] Dewari PS (2018). An efficient and scalable pipeline for epitope tagging in mammalian stem cells using Cas9 ribonucleoprotein. eLife.

[31] Einhauer A, Jungbauer A (2001). The FLAG^TM^ peptide, a versatile fusion tag for the purification of recombinant proteins. J. Biochem. Biophys. Methods.

[32] Hopp TP (1988). A short polypeptide marker sequence useful for recombinant protein identification and purification. Bio/Technology.

[33] Field J (1988). Purification of a RAS-responsive adenylyl cyclase complex from *Saccharomyces cerevisiae* by use of an epitope addition method. Mol. Cell. Biol..

[34] Southern Ja, Young DF, Heaney F, Baumgartner WK, Randall RE (1991). Identification of an epitope on the P and V proteins of simian virus 5 that distinguishes between two isolates with different biological characteristics. J. Gen. Virol..

[35] Fujii Y (2014). PA tag: A versatile protein tagging system using a super high affinity antibody against a dodecapeptide derived from human podoplanin. Protein Expr. Purif..

[36] Bastin P, Bagherzadeh A, Matthews KR, Gull K (1996). A novel epitope tag system to study protein targeting and organelle biogenesis in *Trypanosoma brucei*. Mol. Biochem. Parasitol..

[37] Lobbestael E (2010). Immunohistochemical detection of transgene expression in the brain using small epitope tags. BMC Biotechnol..

[38] Brinkman, A. B. & Stunnenberg, H. G. Strategies for epigenome analysis in *Epigenomics* 3–18 (Springer Netherlands, 2009).

[39] Luo S, Wehr NB, Levine RL (2006). Quantitation of protein on gels and blots by infrared fluorescence of Coomassie blue and Fast Green. Anal. Biochem..

[40] Collett MS, Erikson RL (1978). Protein kinase activity associated with the avian sarcoma virus *src* gene product. Proc. Natl. Acad. Sci. USA.

[41] Zhang L, Rayner S, Katoku-Kikyo N, Romanova L, Kikyo N (2007). Successful co-immunoprecipitation of Oct4 and Nanog using cross-linking. Biochem. Biophys. Res. Commun..

[42] Pollard TD (2010). A guide to simple and informative binding assays. Mol. Biol. Cell.

[43] Cristea IM, Williams R, Chait BT, Rout MP (2005). Fluorescent proteins as proteomic probes. Mol. Cell. Proteomics.

[44] Burbelo PD, Goldman R, Mattson TL (2005). A simplified immunoprecipitation method for quantitatively measuring antibody responses in clinical sera samples by using mammalian-produced Renilla luciferase-antigen fusion proteins. BMC Biotechnol..

[45] Zhang H, Williams PS, Zborowski M, Chalmers JJ (2006). Binding affinities/avidities of antibody–antigen interactions: Quantification and scale-up implications. Biotechnol. Bioeng..

[46] Goldberg ME, Djavadi-Ohaniance L (1993). Methods for measurement of antibody/antigen affinity based on ELISA and RIA. Curr. Opin. Immunol..

[47] Kimura H, Hayashi-Takanaka Y, Goto Y, Takizawa N, Nozaki N (2008). The organization of histone H3 modifications as revealed by a panel of specific monoclonal antibodies. Cell Struct. Funct..

[48] Braunstein M, Rose AB, Holmes SG, Allis CD, Broach JR (1993). Transcriptional silencing in yeast is associated with reduced nucleosome acetylation. Genes Dev..

[49] Gentsch GE, Patrushev I, Smith JC (2015). Genome-wide snapshot of chromatin regulators and states in *Xenopus* embryos by ChIP-Seq. J. Vis. Exp..

[50] Dahl JA, Collas P (2007). Q^2^ ChIP, a quick and quantitative chromatin Immunoprecipitation assay, unravels epigenetic dynamics of developmentally regulated genes in human carcinoma cells. Stem Cells.

[51] Wegner GJ, Lee HJ, Corn RM (2002). Characterization and optimization of peptide arrays for the study of epitope−antibody interactions using surface plasmon resonance imaging. Anal. Chem..

[52] Firsov D (1996). Cell surface expression of the epithelial Na channel and a mutant causing Liddle syndrome: A quantitative approach. Proc. Natl. Acad. Sci. USA.

[53] Park SH (2008). Generation and application of new rat monoclonal antibodies against synthetic FLAG and OLLAS tags for improved immunodetection. J. Immunol. Methods.

[54] Honey S (2001). A novel multiple affinity purification tag and its use in identification of proteins associated with a cyclin-CDK complex. Nucleic Acids Res..

[55] Domanski M (2012). Improved methodology for the affinity isolation of human protein complexes expressed at near endogenous levels. Biotechniques.

[56] Hernan R, Heuermann K, Brizzard B (2000). Multiple epitope tagging of expressed proteins for enhanced detection. Biotechniques.

[57] Kemmer G, Keller S (2010). Nonlinear least-squares data fitting in Excel spreadsheets. Nat. Protoc..

[58] Glass TR (2004). Development and characterization of new monoclonal antibodies specific for coplanar polychlorinated biphenyls. Anal. Chim. Acta.

[59] Nishikori S (2012). Broad ranges of affinity and specificity of anti-histone antibodies revealed by a quantitative peptide immunoprecipitation assay. Journal of Molecular Biology.

[60] Hattori T (2013). Recombinant antibodies to histone post-translational modifications. Nat. Methods.

[61] Drake AW, Myszka DG, Klakamp SL (2004). Characterizing high-affinity antigen/antibody complexes by kinetic- and equilibrium-based methods. Anal. Biochem..

[62] Sosnick TR, Benjamin DC, Novotny J, Seeger PA, Trewhella J (1992). Distances between the antigen-binding sites of three murine antibody subclasses measured using neutron and x-ray scattering. Biochemistry.

[63] Zhang L, Hernan R, Brizzard B (2001). Multiple tandem epitope tagging for enhanced detection of protein expressed in mammalian cells. Mol. Biotechnol..

[64] Marcon E (2015). Assessment of a method to characterize antibody selectivity and specificity for use in immunoprecipitation. Nat. Methods.

[65] Hakhverdyan Z (2015). Rapid, optimized interactomic screening. Nat. Methods.

[66] Wilkinson, K. D. Quantitative analysis of protein–protein interactions in *Methods in Molecular Biology* **261**: *Protein-Protein Interactions*, 15–32 (Humana Press, 2004). 10.1385/1-59259-762-9:015 15064447

[67] Link V, Shevchenko A, Heisenberg CP (2006). Proteomics of early zebrafish embryos. BMC Dev. Biol..

