# Supplementary material for: HiBiT-qIP, HiBiT-based quantitative immunoprecipitation, facilitates the determination of antibody affinity under immunoprecipitation conditions
Source: Sci Rep. 2019 May 3;9:6895. doi: 10.1038/s41598-019-43319-y (PMC6499798; doi:10.1038/s41598-019-43319-y)
Supplement: Supplementary file 1 — Supplementary Information [file 41598_2019_43319_MOESM1_ESM.pdf]

Supplementary Information

HiBiT-qIP, HiBiT-based quantitative immunoprecipitation, facilitates the determination of antibody affinity under immunoprecipitation conditions

Deshani C. Ranawakage, Takuya Takada and Yusuke Kamachi

Figure S1: Nucleotide and amino acid sequences of composite epitope tags

```
(A)
1<----------FLAG---------------->---------<----------TEV------------>---------<-------------------Bio tag------------------------------->---------
GAT TAT AAA GAC GAC GAT GAT AAG ACG CGT GAG AAC CTG TAC TTC CAG GGT GGA GGG GGA CTC AAT GAC ATT TTT GAA GCT CAA AAG ATC GAG TGG CAC GAG GGC GCT
 D   Y   K   D   D   D   D   K   T   R   E   N   L   Y   F   Q   G   G   G   G   L   N   D   I   F   E   A   Q   K   I   E   W   H   E   G   A
<-----------------HiBiT------------------->-144
GTG AGC GGC TGG CGG CTG TTC AAG AAG ATT AGC TAA
 V   S   G   W   R   L   F   K   K   I   S   *
(B)
1<-----------------------------------------------FLAGx3------------------------------------------------>---------<----------TEV------------>----
GAT TAT AAA GAC GAC GAT GAC AAA GGA GAC TAC AAG GAC GAT GAC GAC AAA ATT GAT TAC AAG GAC GAT GAT GAT AAG ACG CGT GAG AAC CTG TAC TTC CAG GGT GGA
 D   Y   K   D   D   D   D   K   G   D   Y   K   D   D   D   D   K   I   D   Y   K   D   D   D   D   K   T   R   E   N   L   Y   F   Q   G   G
-----<------------------Bio tag-------------------------------->---------<-----------------HiBiT------------------>-198
GGG GGA CTC AAT GAC ATT TTT GAA GCT CAA AAG ATC GAG TGG CAC GAG GGC GCT GTG AGC GGC TGG CGG CTG TTC AAG AAG ATT AGC TAA
 G   G   L   N   D   I   F   E   A   Q   K   I   E   W   H   E   G   A   V   S   G   W   R   L   F   K   K   I   S   *
(C)
1<--------------HA----------------->---------<----------TEV------------>---------<-------------------Bio tag------------------------------->----
TAT CCG TAT GAT GTG CCT GAT TAC GCT ACG CGT GAG AAC CTG TAC TTC CAG GGT GGA GGG GGA CTC AAT GAC ATT TTT GAA GCT CAA AAG ATC GAG TGG CAC GAG GGC
 Y   P   Y   D   V   P   D   Y   A   T   R   E   N   L   Y   F   Q   G   G   G   G   L   N   D   I   F   E   A   Q   K   I   E   W   H   E   G
-----<----------------HiBiT------------------->-147
GCT GTG AGC GGC TGG CGG CTG TTC AAG AAG ATT AGC TAA
 A   V   S   G   W   R   L   F   K   K   I   S   *
(D)
1<----------------------------------------------------HAx3------------------------------------------------------>---------<----------TEV-----
TAT CCG TAT GAT GTG CCT GAT TAC GCT GGC TAC CCC TAC GAT GTC CCA GAC TAC GCC GGA TAC CCT TAT GAC GTT CCA GAC TAT GCA ACG CGT GAG AAC CTG TAC TTC
 Y   P   Y   D   V   P   D   Y   A   G   Y   P   Y   D   V   P   D   Y   A   G   Y   P   Y   D   V   P   D   Y   A   T   R   E   N   L   Y   F
------->---------<-------------------Bio tag-------------------------------->---------<----------------HiBiT------------------>-147
CAG GGT GGA GGG GGA CTC AAT GAC ATT TTT GAA GCT CAA AAG ATC GAG TGG CAC GAG GGC GCT GTG AGC GGC TGG CGG CTG TTC AAG AAG ATT AGC TAA
 Q   G   G   G   G   L   N   D   I   F   E   A   Q   K   I   E   W   H   E   G   A   V   S   G   W   R   L   F   K   K   I   S   *
(E)
1<-------------------PA--------------------->---------<----------TEV------------>---------<-------------------Bio tag------------------------
GGC GTT GCC ATG CCA GGT GCC GAA GAT GAT GTG GTG ACG CGT GAG AAC CTG TAC TTC CAG GGT GGA GGG GGA CTC AAT GAC ATT TTT GAA GCT CAA AAG ATC GAG TGG
 G   V   A   M   P   G   A   E   D   D   V   V   T   R   E   N   L   Y   F   Q   G   G   G   G   L   N   D   I   F   E   A   Q   K   I   E   W
------->--------<-----------------HiBiT------------------>-156
CAC GAG GGC GCT GTG AGC GGC TGG CGG CTG TTC AAG AAG ATT AGC TAA
 H   E   G   A   V   S   G   W   R   L   F   K   K   I   S   *
```

Figure S1: Nucleotide and amino acid sequences of composite epitope tags

```
(F)
1<------------------------------------------------PAx2-------------------------------------------->---------<----------TEV------------>--------
GGG GTT GCA ATG CCT GGT GCT GAG GAT GAT GTC GTG GGC GGA GTG GCT ATG CCA GGC GCG GAA GAC GAC GTA GTG ACG CGT GAG AAC CTG TAC TTC CAG GGT GGA GGG
G   V   A   M   P   G   A   E   D   D   V   V   G   G   V   A   M   P   G   A   E   D   D   V   V   T   R   E   N   L   Y   F   Q   G   G   G
-<-----------------Bio tag-------------------------------->---------<------------------HiBiT------------------->-195
GGA CTC AAT GAC ATT TTT GAA GCT CAA AAG ATC GAG TGG CAC GAG GGC GCT GTG AGC GGC TGG CGG CTG TTC AAG AAG ATT AGC TAA
 G   L   N   D   I   F   E   A   Q   K   I   E   W   H   E   G   A   V   S   G   W   R   L   F   K   K   I   S   *
(G)
1<---------------------------------------------------------------------PAx3------------------------------------------------------------------------
GGG GTT GCA ATG CCT GGT GCT GAG GAT GAT GTC GTG GGC GGA GTG GCT ATG CCA GGC GCG GAA GAC GAC GTA GTG ACG CGC GGC GTT GCC ATG CCA GGT GCC GAA GAT
 G   V   A   M   P   G   A   E   D   D   V   V   G   G   V   A   M   P   G   A   E   D   D   V   V   T   R   G   V   A   M   P   G   A   E   D
---------->---------<----------TEV------------>---------<-----------------------Bio tag--------------------------->---------<------------------
GAT GTG GTG ACG CGT GAG AAC CTG TAC TTC CAG GGT GGA GGG GGA CTC AAT GAC ATT TTT GAA GCT CAA AAG ATC GAG TGG CAC GAG GGC GCT GTG AGC GGC TGG CGG
 D   V   V   T   R   E   N   L   Y   F   Q   G   G   G   G   L   N   D   I   F   E   A   Q   K   I   E   W   H   E   G   A   V   S   G   W   R
HiBiT--------------------237
CTG TTC AAG AAG ATT AGC TAA
 L   F   K   K   I   S   *
(H)
1<--------------------V5------------------------------>---------<----------TEV------------>---------<-------------------Bio tag----------------
GGT AAG CCC ATT CCA AAC CCA CTC CTG GGG TTA GAC TCT ACC ACG CGT GAG AAC CTG TAC TTC CAG GGT GGA GGG GGA CTC AAT GAC ATT TTT GAA GCT CAA AAG ATC
 G   K   P   I   P   N   P   L   L   G   L   D   S   T   T   R   E   N   L   Y   F   Q   G   G   G   L   N   D   I   F   E   A   Q   K   I
--------------> --------<-----------------HiBiT------------------->-162
GAG TGG CAC GAG GGC GCT GTG AGC GGC TGG CGG CTG TTC AAG AAG ATT AGC TAA
 E   W   H   E   G   A   V   S   G   W   R   L   F   K   K   I   S   *
(I)
1<------------------------------------------------V5x2------------------------------------------------------------>---------<----------TEV-----
GGT AAG CCC ATT CCA AAC CCA CTC CTG GGG TTA GAC TCT ACC GGA GGC AAA CCG ATC CCT AAT CCT CTT TTG GGA CTG GAT AGC ACA ACG CGT GAG AAC CTG TAC TTC
G   K   P   I   P   N   P   L   L   G   L   D   S   T   G   G   K   P   I   P   N   P   L   L   G   L   D   S   T   T   R   E   N   L   Y   F
------->---------<-----------------Bio tag-------------------------------->---------<-----------------HiBiT------------------->-207
CAG GGT GGA GGG GGA CTC AAT GAC ATT TTT GAA GCT CAA AAG ATC GAG TGG CAC GAG GGC GCT GTG AGC GGC TGG CGG CTG TTC AAG AAG ATT AGC TAA
 Q   G   G   G   G   L   N   D   I   F   E   A   Q   K   I   E   W   H   E   G   A   V   S   G   W   R   L   F   K   K   I   S   *
```

Figure S1: Nucleotide and amino acid sequences of composite epitope tags

```
(J)
1<------------------------------------------------------------------------V5x3-----------------------------------------------------------------
GGA AAG CCA ATC CCG AAT CCC CTT CTT GGA CTC GAC TCC ACA GGA GGT AAG CCC ATT CCA AAC CCA CTC CTG GGG TTA GAC TCT ACC GGA GGC AAA CCG ATC CCT AAT
G   K   P   I   P   N   P   L   L   G   L   D   S   T   G   G   K   P   I   P   N   P   L   L   G   L   D   S   T   G   G   K   P   I   P   N
------------------------------>---------<----------TEV------------>---------<-----------------Bio tag--------------------------------->--------
CCT CTT TTG GGA CTG GAT AGC ACA ACG CGT GAG AAC CTG TAC TTC CAG GGT GGA GGG GGA CTC AAT GAC ATT TTT GAA GCT CAA AAG ATC GAG TGG CAC GAG GGC GCT
 P   L   L   G   L   D   S   T   T   R   E   N   L   Y   F   Q   G   G   G   G   L   N   D   I   F   E   A   Q   K   I   E   W   H   E   G   A
-<----------------HiBiT------------------->-252
GTG AGC GGC TGG CGG CTG TTC AAG AAG ATT AGC TAA
 V   S   G   W   R   L   F   K   K   I   S   *
(K)
1<--------------Ty1------------------->---------<----------TEV------------>---------<-------------------Bio tag-------------------------------
GAG GTG CAC ACT AAT CAA GAT CCT CTG GAC ACG CGT GAG AAC CTG TAC TTC CAG GGT GGA GGG GGA CTC AAT GAC ATT TTT GAA GCT CAA AAG ATC GAG TGG CAC GAG
E   V   H   T   N   Q   D   P   L   D   T   R   E   N   L   Y   F   Q   G   G   G   G   L   N   D   I   F   E   A   Q   K   I   E   W   H   E
>--------<-----------------HiBiT------------------->-150
GGC GCT GTG AGC GGC TGG CGG CTG TTC AAG AAG ATT AGC TAA
 G   A   V   S   G   W   R   L   F   K   K   I   S   *
(L)
1<--------------------------------------Ty1X2------------------------------------->---------<----------TEV------------>---------<--------------
GAG GTG CAT ACA AAT CAG GAC CCT CTC GAT GCT GAA GTC CAC ACC AAC CAA GAT CCA CTG GAC ACG CGT GAG AAC CTG TAC TTC CAG GGT GGA GGG GGA CTC AAT GAC
E   V   H   T   N   Q   D   P   L   D   A   E   V   H   T   N   Q   D   P   L   D   T   R   E   N   L   Y   F   Q   G   G   G   G   L   N   D
-----Biotag------------------------------->---------<-----------------HiBiT------------------->-183
ATT TTT GAA GCT CAA AAG ATC GAG TGG CAC GAG GGC GCT GTG AGC GGC TGG CGG CTG TTC AAG AAG ATT AGC TAA
 I   F   E   A   Q   K   I   E   W   H   E   G   A   V   S   G   W   R   L   F   K   K   I   S   *
(M)
1<--------------------------------------------------------------Ty1X3------------------------------------------------------------->---------<--
GAG GTG CAT ACA AAT CAG GAC CCT CTC GAT GCT GAA GTC CAC ACC AAC CAA GAT CCA CTG GAC ACG CGC GAG GTG CAC ACT AAT CAA GAT CCT CTG GAC ACG CGT GAG
 E   V   H   T   N   Q   D   P   L   D   A   E   V   H   T   N   Q   D   P   L   D   T   R   E   V   H   T   N   Q   D   P   L   D   T   R   E
--------TEV------------>---------<-------------------Biotag------------------------------->---------<-----------------HiBiT-------------------
AAC CTG TAC TTC CAG GGT GGA GGG GGA CTC AAT GAC ATT TTT GAA GCT CAA AAG ATC GAG TGG CAC GAG GGC GCT GTG AGC GGC TGG CGG CTG TTC AAG AAG ATT AGC
 N   L   Y   F   Q   G   G   G   G   L   N   D   I   F   E   A   Q   K   I   E   W   H   E   G   A   V   S   G   W   R   L   F   K   K   I   S
>-219
TAA
 *
```

Supplementary Figure 1. Nucleotide and amino acid sequences of the composite epitope tags. The amino acid sequences of the composite epitope tags are shown with the corresponding nucleotide sequences. Each nucleotide sequence is preceded by the *Xho*I site sequence CTCGAG and followed by the *Xba*I-*Not*I linker sequence TCTAGAGCGGCCGC for cloning into pGEX-6P-1. TEV, TEV protease cleavage site; Bio tag, biotin ligase recognition site.

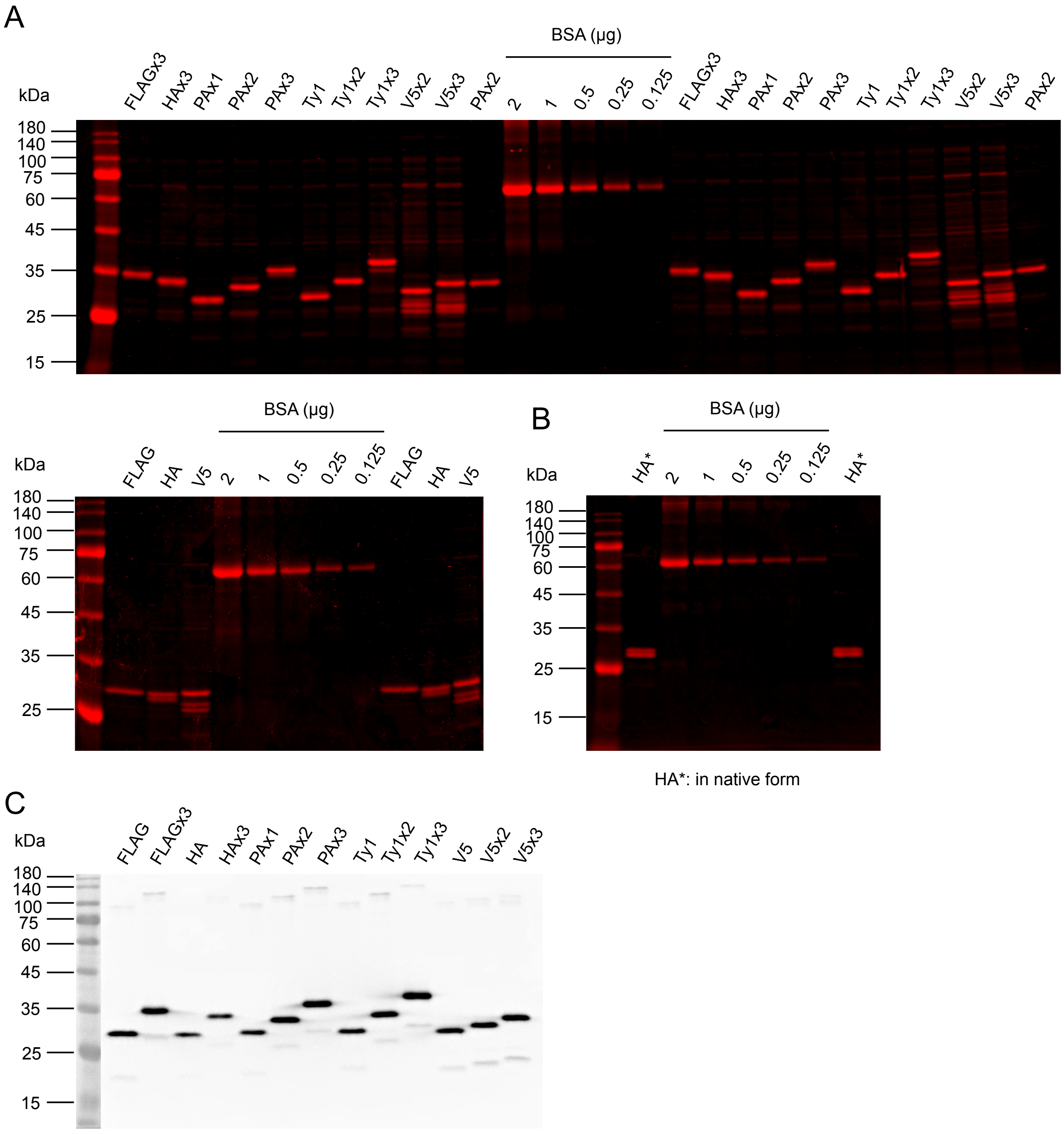

Supplementary Figure 2. Quantification of GST-epitope tag fusion protein. (**A**) SDS polyacrylamide gel images of GST proteins fused with monomeric, dimeric and trimeric forms of the epitope tags, which were prepared in denatured form. Each GST fusion was loaded in duplicate, and BSA standards were loaded for quantification. The gels were stained with Coomassie Brilliant Blue G-250 and detected by near-infrared fluorescence. (**B**) SDS polyacrylamide gel image of the GST protein fused with a monomeric form of the HA epitope tag, which was prepared in native form. (**C**) HiBiT blot detection of the series of tagged GST proteins to confirm retention of the C-terminal HiBiT tag.

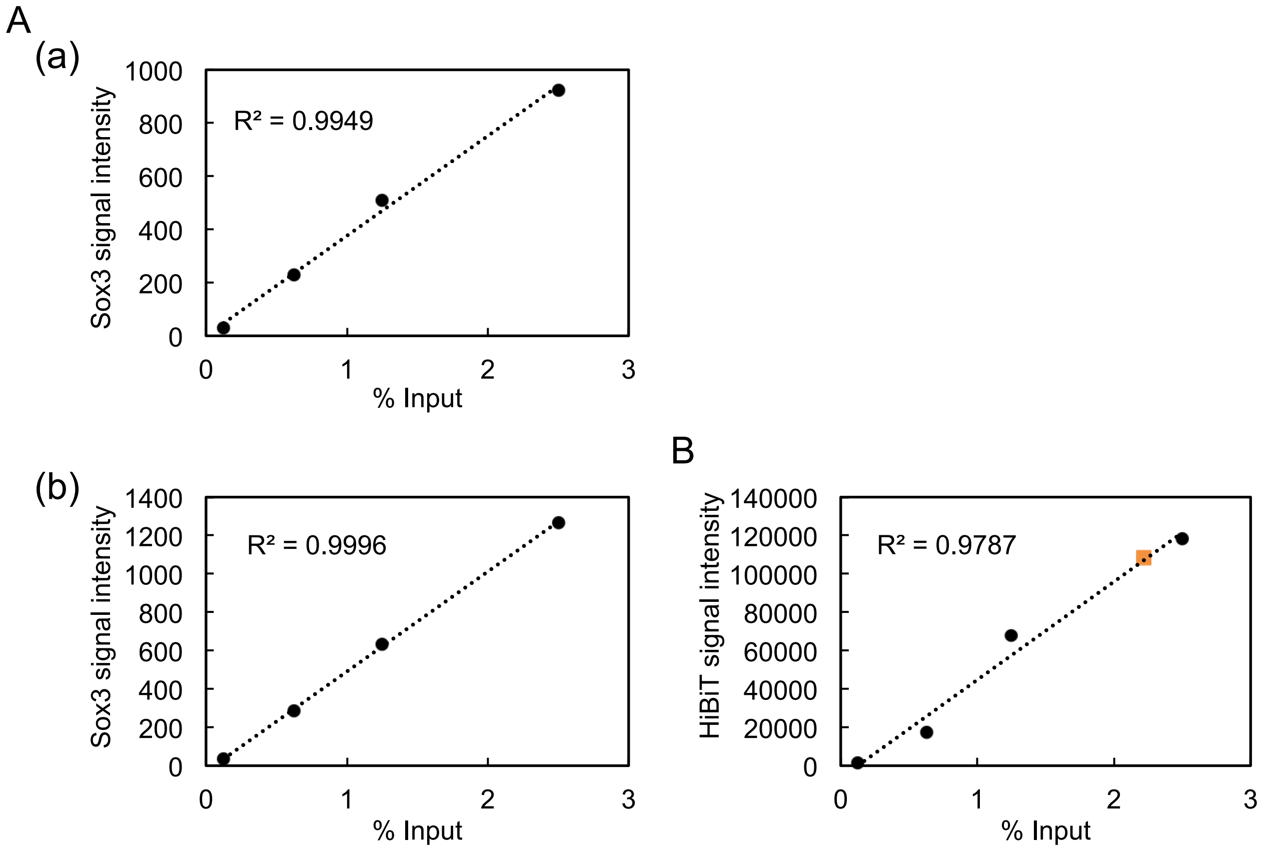

Supplementary Figure 3. Standard curves demonstrating the linear correlation of the signal intensity with the percent input. (**A**) The near-infrared fluorescence (NIR) signals were linear across the entire range of percent inputs tested, with $R^2 > 0.99$ for the Sox3 protein tagged with FLAG in monomeric (a) or trimeric (b) form. (**B**) The HiBiT-derived bioluminescence was linear across the entire range of percent inputs tested, with $R^2 > 0.97$ for the FLAGx3-tagged-Sox3 protein. The signal intensity for 0.5 ng of FLAGx3-tagged GST was plotted on the calibration curve (orange square).

**Epitope tag antibody**	**K_d (nM)**	**Assay format/ method**	**References**
FLAG (M2)	0.76	HiBiT-qIP	This study
	28	SPR	Fujii et al. (2014) [35]
	6.7	SPR	Wegner et al. (2002) [51]
	3	Saturation binding and Scatchard analysis	Firsov et al. (1996)[52]
HA (4B2)	6.6	HiBiT-qIP	This study
	1.6	SPR	Fujii et al. (2014) [35]
PA (NZ-1)	0.65	HiBiT-qIP	This study
	0.40	SPR	Fujii et al. (2014) [35]

Supplementary Table 2. Comparison of the K_d values obtained in this study with those reported in the literature. To the best of our knowledge, all the available K_d values for the interactions of monoclonal antibodies with the epitope tags used in this study are listed in this table.
